# The bZIP73 transcription factor controls rice cold tolerance at the reproductive stage

**DOI:** 10.1111/pbi.13104

**Published:** 2019-03-12

**Authors:** Citao Liu, Michael R. Schläppi, Bigang Mao, Wei Wang, Aiju Wang, Chengcai Chu

**Affiliations:** ^1^ State Key Laboratory of Plant Genomics, Institute of Genetics and Developmental Biology Chinese Academy of Sciences Beijing China; ^2^ Department of Biological Sciences Marquette University Milwaukee WI USA; ^3^ State Key Laboratory of Hybrid Rice China National Hybrid Rice Research and Development Center Changsha China; ^4^ University of Chinese Academy of Sciences Beijing China

**Keywords:** cold tolerance, *bZIP73^Jap^*, ABA level, sugar transport, reproductive stage

## Abstract

Cold temperature during the reproductive stage often causes great yield loss of grain crops in subtropical and temperate regions. Previously we showed that the rice transcription factor bZIP73^Jap^ plays an important role in cold adaptation at the seedling stage. Here we further demonstrate that bZIP73^Jap^ also confers cold stress tolerance at the reproductive stage. *bZIP73* ^*Jap*^ was up‐regulated under cold treatment and predominately expressed in panicles at the early binucleate and flowering stages. bZIP73^Jap^ forms heterodimers with bZIP71, and co‐expression of *bZIP73* ^*Jap*^ and *bZIP71* transgenic lines significantly increased seed‐setting rate and grain yield under natural cold stress conditions. bZIP73^Jap^:bZIP71 not only repressed ABA level in anthers, but also enhanced soluble sugar transport from anthers to pollens and improved pollen grain fertility, seed‐setting rate, and grain yield. Interestingly, bZIP73^Jap^:bZIP71 also regulated the expression of *qLTG3‐1* ^*Nip*^, and *qLTG3‐1* ^*Nip*^ overexpression lines greatly improved rice tolerance to cold stress during the reproductive stage. Therefore, our work establishes a framework for rice cold stress tolerance through the bZIP71‐bZIP73^Jap^‐qLTG3‐1^Nip^‐sugar transport pathway. Together with our previous work, our results provide a powerful tool for improving rice cold stress tolerance at both the seedling and the reproductive stages.

## Introduction

As rice originated from tropical and subtropical regions, it is vulnerable to cold stress at all growth stages, which is especially critical during the reproductive stage (booting and flowering stage), because it adversely affects grain yield and quality (Espe *et al*., 2017; Pan *et al*., 2015; Zhang *et al*., 2014, 2017). During different booting stages, including the transition of the tetrad to early uni‐nucleate stage (also called young microspore or YM stage), and the early binucleate (EB) stage, cold stress may cause sterile pollen grains or reduced number of mature pollen grains, especially at the YM stage, thus leading to spikelet sterility (Oliver *et al*., 2005; Satake, 1976; Shimono *et al*., 2016; Suh *et al*., 2010). During the flowering stage, cold stress may affect pollen germination, pollen tube elongation, fertilization, also leading to spikelet sterility (Shinada *et al*., 2013, 2014). Rice is widely planted in China, from Hainan island (18°90′N) to the Mohe River (53°27′N) in Heilongjiang, and from the eastern coastal areas to the Yunnan‐Guizhou Plateau and Xinjiang in Western China, and almost all rice production areas are highly vulnerable to cold injury, leading to an estimated annual loss of about 3–5 million tons in China (Li *et al*., 2018; Liu *et al*., 2018b; Zhang *et al*., 2017; Zhu *et al*., 2015). Therefore, breeding cold‐tolerant varieties is an effective method to maintain high and stable yields in those rice cultivation regions.

Cold tolerance is a quantitative trait that is controlled by multiple loci and also affected by the environment (Li *et al*., 2018; Sun *et al*., 2018; Zhang *et al*., 2017). Over the past decades, only three QTL, *CTB4a*,* Ctb1* and *qPSR10*, have been cloned and functionally characterized (Saito *et al*., 2010; Xiao *et al*., 2018; Zhang *et al*., 2017). *CTB4a* encodes a conserved leucine‐rich repeat receptor‐like kinase that interacts with AtpB, thus increasing ATP synthase activity and ATP content and enhancing seed‐setting rate and improved yield under cold stress conditions (Zhang *et al*., 2017). *Ctb1* encodes an F‐box protein, suggesting that a ubiquitin‐proteasome pathway is involved in chilling tolerance at this stage (Saito *et al*., 2010). *qPSR10* was identified by GWAS using a 1033‐accession diversity panel and confers cold tolerance both at the seedling and reproductive stages (Xiao *et al*., 2018). However, little is known about the molecular mechanisms of rice cold tolerance at the reproductive stage.

Morphological and histological investigations indicated that cold results in reduced number of mature pollen grains, thus increasing the rate of male sterility (Nishiyama, 1976, 1982; Sakata *et al*., 2014). The proposed mechanism for cold‐induced pollen sterility (CIPS) is that cold leads to an absence of starch accumulation in mature pollen grains and concurrent accumulation of sugar in anthers due to repression of anther cell wall invertase and monosaccharide transporter genes, thus resulting in reduced pollen sink strength (Ji *et al*., 2011; Koonjul *et al*., 2005; Oliver *et al*., 2005). In agreement with this, expression of cell wall invertase and monosaccharide transporter genes is negatively affected under cold stress conditions (Ji *et al*., 2011; Oliver *et al*., 2007; Sakata *et al*., 2014).

Beijing is located in Northern China (39.9°N) where low temperature stress during the rice growing season often occurs. Previously, we reported that *bZIP73* ^*Jap*^, the *japonica* version of *bZIP73*, a bZIP transcription factor‐coding gene with only one functional polymorphism (+511 G>A) between the two subspecies *japonica* and *indica*, significantly improved rice tolerance to cold stress at the seedling stage when co‐expressed with *bZIP71* (Liu *et al*., 2018a). Here, we further demonstrate that bZIP71 and bZIP73^Jap^ also improve rice cold tolerance at the reproductive stage by mediating ABA and sugar partitioning in flowers, which indicates that these factors can improve cold tolerance throughout the entire growth period of rice.

## Results

### Expression patterns of *bZIP73* ^*Jap*^ and *bZIP71*

**Figure 1:**
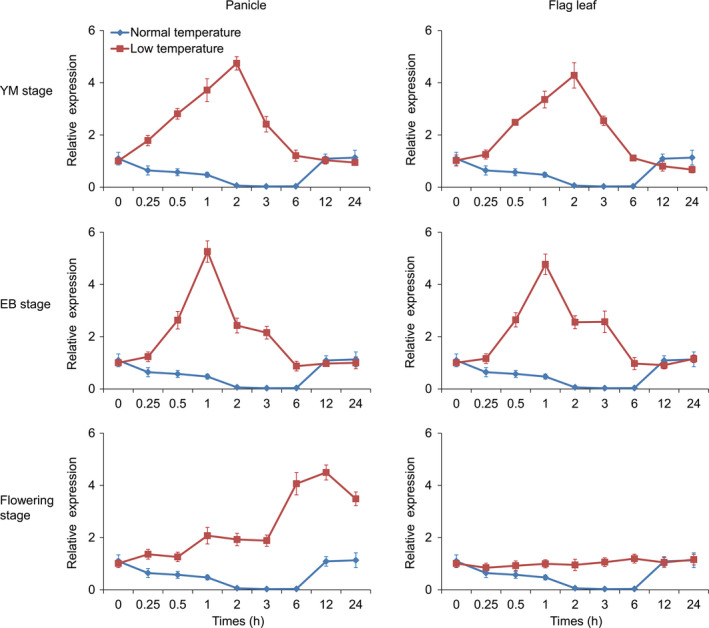
*bZIP73* ^*Jap*^ expression patterns in flag leaves and panicles of wild‐type ZH11 under cold treatment during the reproductive stage. YM, young microspore stage. EB, early binucleate stage. Plants grown under normal warm temperature conditions were used as untreated control. Error bar, standard deviation from three independent experiments.

Previously we demonstrated that the bZIP71:bZIP73^Jap^ heterodimer plays an important role in rice cold adaptation at the seedling stage (Liu *et al*., 2018a). To further elucidate bZIP71:bZIP73^Jap^ functions, the expression of *bZIP71* and *bZIP73* ^*Jap*^ in different tissues was examined. *bZIP73* ^*Jap*^ was predominately expressed in panicles at the early binucleate (EB) and flowering stages (Figure S1), while *bZIP71* was constitutively expressed in almost all tissues and organs examined and its expression was higher in seedlings (shoots and roots) than in other tissues. Further examination of its expression profile under cold stress at the reproductive stage revealed that *bZIP73* ^*Jap*^ was strongly up‐regulated both in panicles and flag leaves following 3 h of cold treatment (Figure 1) and reached the highest expression at 1 h (EB stage) or 2 h (YM stage) after cold treatment (Figure 1). At the flowering stage, *bZIP73* ^*Jap*^ was up‐regulated in panicles during a 24 h time course and reached the highest expression at 12 h after cold treatment. However, *bZIP71* was unchanged after cold treatment at the YM, EB, and flowering stages (Figure S2). These results indicated that *bZIP73* ^*Jap*^ may also participate in cold stress tolerance during the reproductive stage of rice development.

### *bZIP73* ^*Jap*^ and *bZIP71* co‐expression lines significantly improve cold tolerance during the reproduction stage

**Figure 2:**
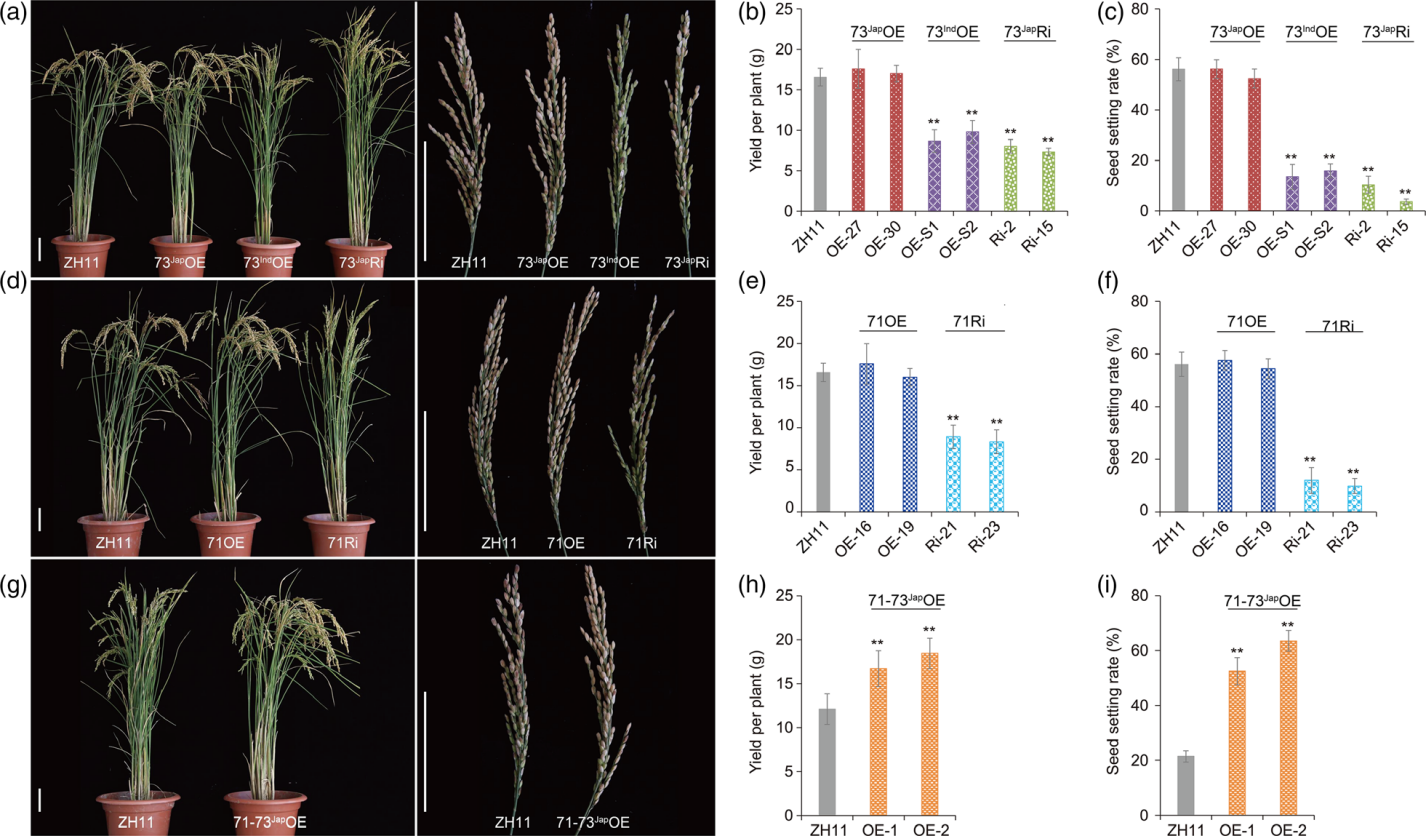
Cold tolerance phenotypes and agronomic traits of *bZIP71* and *bZIP73* transgenic rice plants during the reproductive stage. (a‐c) Overexpression lines *bZIP73* ^*Jap*^ (73^Jap^ OE), *bZIP73* ^*Ind*^ (73^Ind^ OE), and RNAi lines *bZIP73* ^*Jap*^ (73^Jap^Ri) under natural cold stress conditions in Beijing during the autumn of 2012 (heading date: September 13). (b) Yield per plant, (c) Seed‐setting rate. (d–f) *bZIP71* overexpression lines (71OE) and *bZIP71* RNAi (71Ri) lines under natural cold stress conditions in Beijing during the autumn of 2012 (heading date: September 13), (e) Yield per plant, (f) Seed‐setting rate. (g–i) Co‐overexpression lines of *bZIP71*‐*bZIP73* ^*Jap*^ (71‐73^Jap^ OE) under natural cold stress conditions in Beijing during the autumn of 2012 (heading date: September 20), (h) Yield per plant. (i) Seed‐setting rate. Scale bars = 10 cm. All data were collected using two independent homozygous transgenic lines. Three biological replicates (*n* = 30 each) were used for each line. Error bar, standard deviation. **P < 0.01, two‐tailed *t*‐test.

To examine low temperature stress during the reproductive stage under natural growth conditions in Beijing (116.4°E/39.9°N), we adopted a batch sowing approach with different T3 homozygous transgenic lines, including *bZIP73* ^*Jap/Ind*^ overexpression plants (73^Jap^OE, 73^Ind^OE), *bZIP71* overexpression plants (71OE), *bZIP73* ^*Jap*^ RNAi plants (73^Jap^Ri), *bZIP71* RNAi plants (71Ri), and *bZIP73* ^*Jap*^ and *bZIP71* co‐expression plants (71‐73^Jap^OE), and wild‐type Zhonghua 11 (ZH11) as a control. The late sowing rice plants started booting in the time ranging from late August to late September, when the typical night terrestrial temperature was below 20 °C (Figure S3), and agronomic traits were investigated and compared between normal and cold conditions in field plots. In 2012, the late planting transgenic lines, which headed on September 13, met the low temperature stress requirement, experiencing a minimum terrestrial temperature of less than 20 °C during the reproductive stage (Figure S3). The 73^Ind^OE, 73^Jap^Ri, and 71Ri transgenic lines were more sensitive to cold stress with decreased seed‐setting rates and yield per plant compared to wild‐type ZH11, that is, the seed‐setting rate decreased by 40.4%–52.6%, and the yield per plant decreased by 40.8%–55.9% (Figure 2, Table S1). However, the seed‐setting rate and yield per plant of 73^Jap^OE and 71OE lines were not significantly different from those of wild‐type ZH11 plants under natural cold stress. On the other hand, the seed‐setting rate of 71‐73^Jap^OE lines improved by 12%–20.7%, and the yield per plant improved by 40.8%–63.2% (Table S1). Transgenic plants that were planted much later headed on September 20 and also met the low temperature stress requirement, experiencing even lower minimum terrestrial temperatures during the reproductive stage (Figure S3), and the seed‐setting rate of 71‐73^Jap^OE lines improved by 30.7%–42%, while the yield per plant improved by 37.9%–52.1% compared to wild‐type ZH11 (Figure 2, Table S2). Moreover, we found that seed‐setting rate and yield per plant was positively correlated with co‐overexpression levels of *bZIP71* and *bZIP73* ^*Jap*^ (Figure 2, Figure S4). However, we did not observe significant differences for all other agronomic traits between transgenic lines and wild‐type controls under normal temperature conditions (Table S3). These results indicated that co‐overexpression of *bZIP71* and *bZIP73* ^*Jap*^ significantly improves rice tolerance to cold stress during the reproductive period.

### 71‐73^Jap^OE lines increase fertility by increasing the amount of mature pollen grains under cold stress

**Figure 3:**
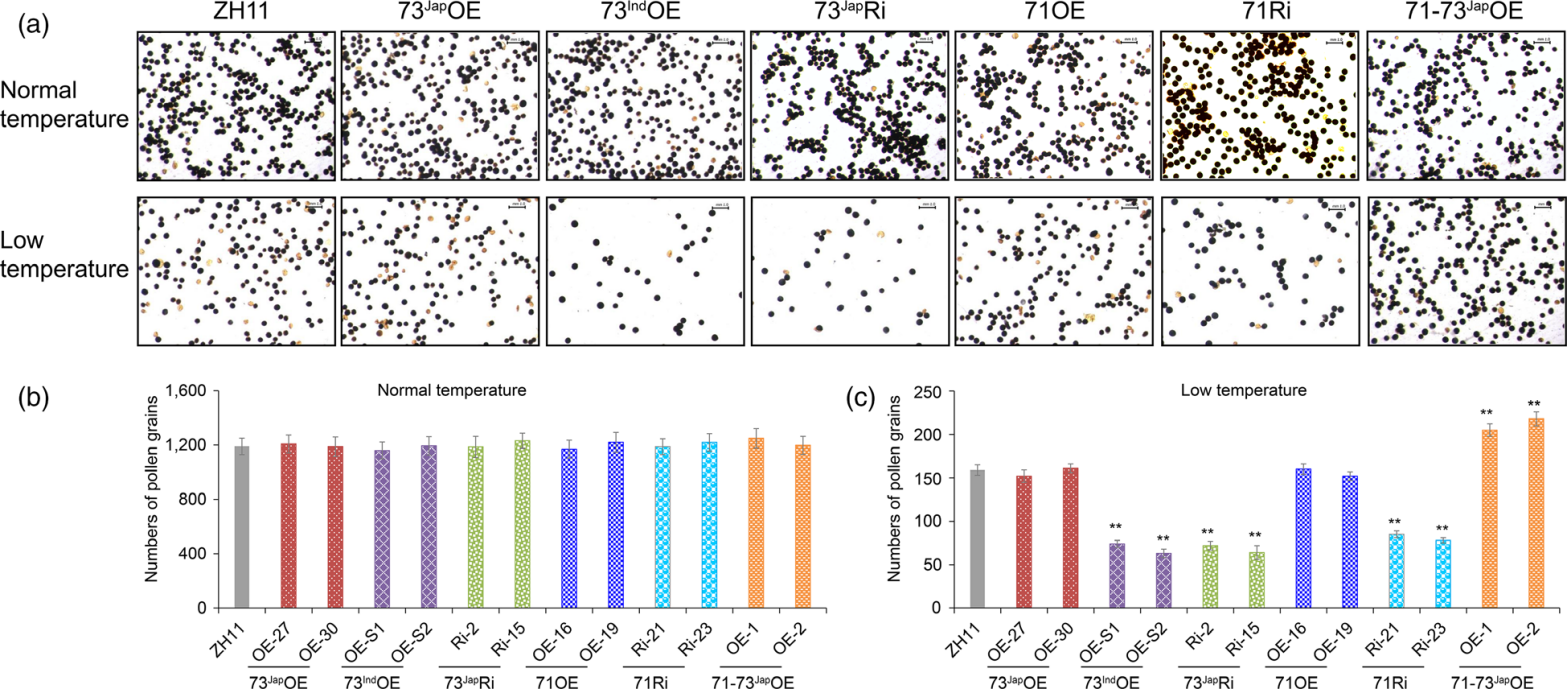
Effects of cold treatment on starch pollen viability and pollen numbers of transgenic lines and ZH11 control plants. (a) KI/I_2_ staining of pollen grains from panicles of transgenic lines and wild‐type ZH11 control plants grown in Beijing, one day before heading either under normal warm temperature conditions (heading date: August 17) or natural cold stress conditions (heading date: September 13) with average terrestrial temperatures below 20 °C (Figure S3). Scale bars = 0.1 mm. Number of pollen grains in anthers of plants grown at warm temperatures (b) and natural cold stress conditions (c). For each plant, three biological replicates, *n* = 3 flowers, were tested. Error bar, standard deviation. **P < 0.01, one way anova test, *P*‐value in Table S8A,B. [Correction added on 16 April 2019, after first online publication. Information regarding Figure 3 was previously incorrect and is updated in this version.]

Rice plants experiencing temperatures lower than 20 °C at the YM stage for only 2–4 successive nights will suffer irreversible pollen sterility and a reduction in pollen number, resulting in reduced fertilization (Mamun *et al*., 2010; Sakata *et al*., 2014; Sharma and Nayyar, 2016). 73^Jap^Ri, 71Ri, and 73^Ind^OE lines had decreased seed‐setting rates, while 71‐73^Jap^OE lines had increased seed‐setting rates compared to wild type ZH11 under cold stress. However, starch staining showed that the rate of pollen sterility in 73^Jap^Ri, 71Ri, and 73^Ind^OE lines was not significantly different compared to wild‐type ZH11 pollen under cold stress (Figure 3a), but compared to wild‐type ZH11, the number of mature pollen grains in those lines was significantly reduced by 46.5%–60.4% under cold stress (Figure 3c). In contrast, the number of mature pollen grains in 71‐73^Jap^OE lines was 28.9%–37.1% higher than in wild‐type under cold stress (Figure 3c), and no differences in the rate of pollen sterility and pollen number between 71‐73^Jap^OE lines and wild‐type was observed under normal growth temperature (Figure 3a,b).

### bZIP71 enhances bZIP73^Jap^ by repressing the expression of ABA synthesis genes and reducing ABA levels in anthers under cold stress

**Figure 4:**
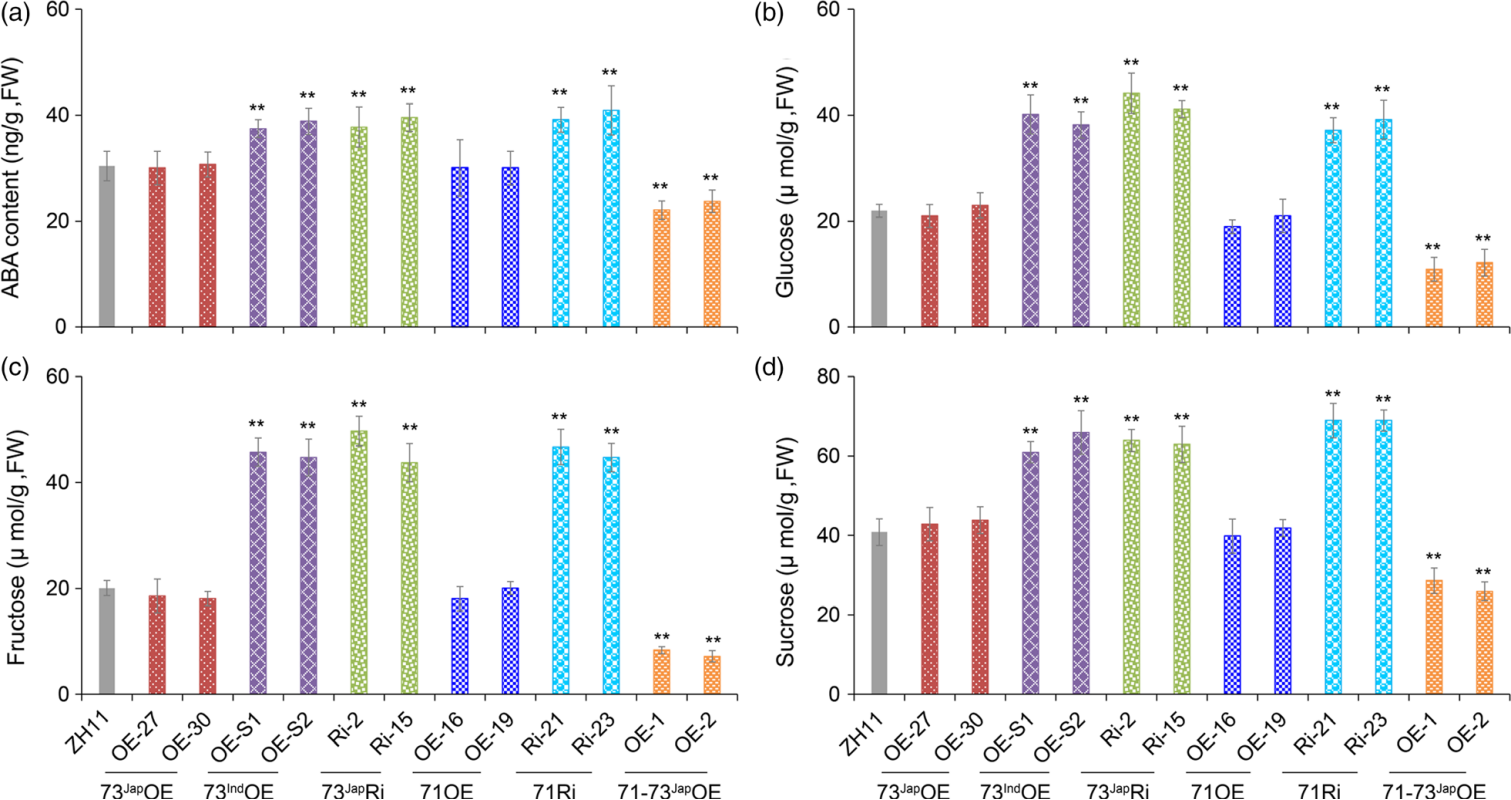
ABA and soluble sugar contents in transgenic lines and wild‐type plants under natural cold stress conditions. Shown are ABA (a), glucose (b), fructose (c), and sucrose (d) contents in transgenic lines and wild‐type ZH11 plants under cold stress conditions. FW, fresh weight. All experiments were conducted using flowers one day before heading. Values are the mean of ten independent biological replicates. Error bars indicate SD. **P < 0.01, one way anova test, *P*‐value in Table S8C–F.

Cold stress leads to abscisic acid (ABA) accumulation in rice anthers (Ji *et al*., 2011; Oliver *et al*., 2007). Our previous work showed that bZIP71 enhances bZIP73^Jap^ by repressing the ABA biosynthesis genes *OsNCED3* and *OsNCED5*, leading to reduced ABA levels in rice seedlings under cold stress (Liu *et al*., 2018a). To test whether *bZIP71* and *bZIP73* ^*Jap*^ also play a role in modulating ABA levels at the reproductive stage, ABA levels in anthers of transgenic lines grown both under normal and low temperature conditions were measured. ABA levels in anthers of 71Ri, 73^Jap^Ri, and 73^Ind^OE lines were 23.1%–34.5% higher than that of wild‐type ZH11 under low temperature conditions (Figure 4a). In contrast, ABA levels in anthers of 71‐73^Jap^OE lines were 22.0%–27.4% lower than in wild‐type ZH11 (Figure 4a). However, ABA levels in anthers of all transgenic lines were not significantly different under normal condition (Figure S5A).

**Figure 5:**
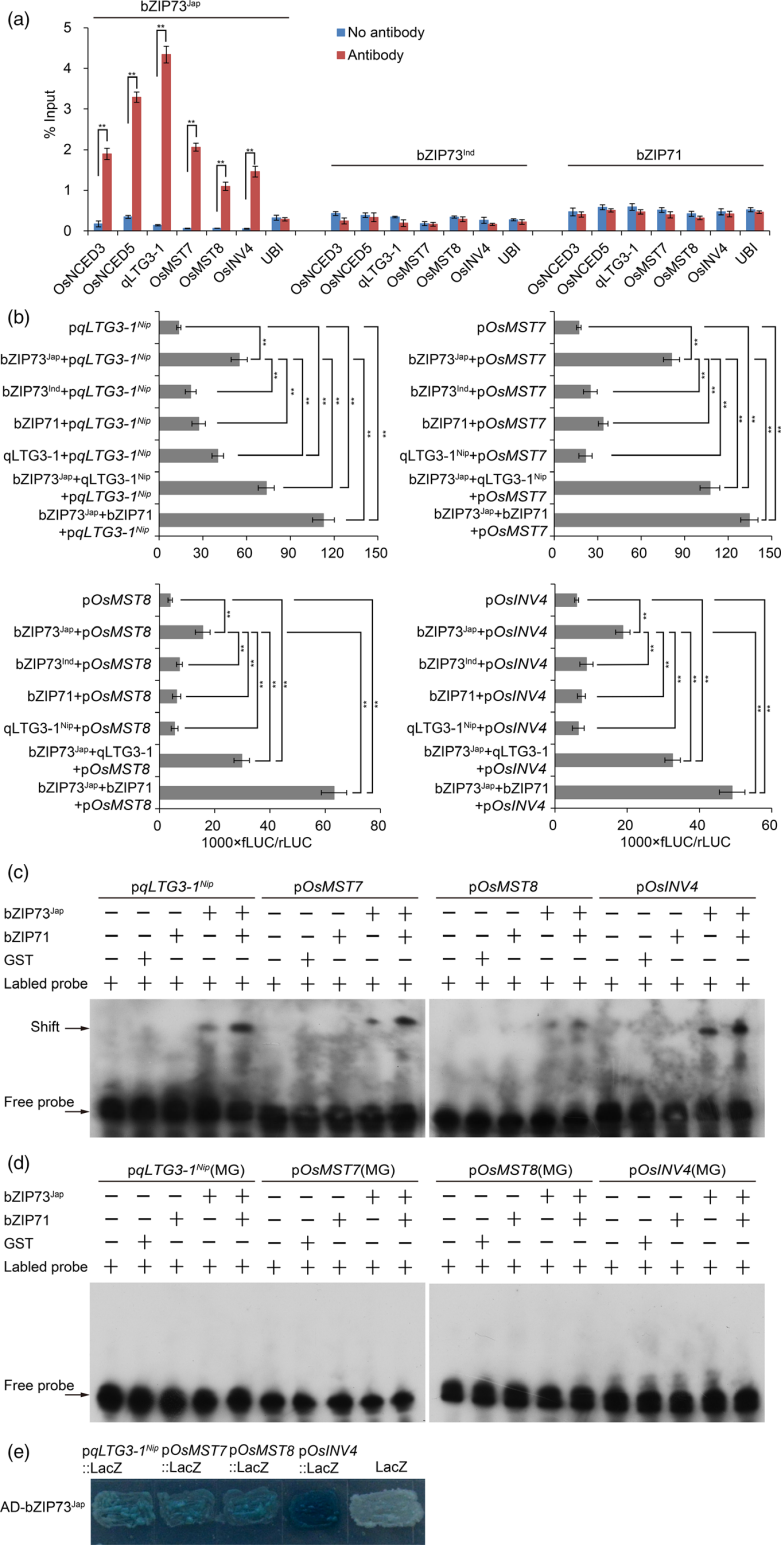
bZIP71 enhances bZIP73^Jap^ *trans*‐activates the expression of *qLTG3‐1* ^*Nip*^, *OsMST7*,* OsMST8* and *OsINV4*. (a) ChIP‐qPCR assays indicating that bZIP73^Jap^ directly binds *in vivo* to promoters of *OsNCED3*,* OsNCED5*, *qLTG3‐1* ^*Nip*^, *OsINV4*,* OsMST7*, and *OsMST8*. Flowers of bZIP73^Jap^::Flag, bZIP73^Ind^::Flag, and bZIP71::Flag overexpression lines were harvested for ChIP analysis using three biological replicates. The anti‐Flag antibody was used to precipitate DNA sequences interacting with different bZIP fusion proteins. Precipitated DNA was amplified with primers overlapping with G‐box motifs. ChIP, chromatin immunoprecipitation. Error bar, standard deviation. **P < 0.01, two‐tailed *t*‐test. (b) Transient *trans*‐activation expression assays in rice protoplasts showing that bZIP71 enhances bZIP73^Jap^ in *trans*‐activating the promoters of *qLTG3‐1* ^*Nip*^, *OsINV4*,* OsMST7* and *OsMST8*. Moreover, qLTG3‐1^Nip^ also enhances bZIP73^Jap^ in *trans*‐activating these downstream genes expression. The relative fLUC/rLUC ratio (see Materials and Methods) shown on the x‐axes represents the relative promoter activity. Constructs used for each transfections into rice protoplasts are shown on the y‐axes. Promoters of *qLTG3‐1* ^*Nip*^, *OsINV4*,* OsMST7*, and *OsMST8* were fused to LUC as the reporter gene. Values are the mean of five independent replicates and error bars indicate SD. **P < 0.01, one way anova test. (c) *In vitro* EMSA showing that bZIP71 promotes bZIP73^Jap^ binding to promoters of *qLTG3‐1* ^*Nip*^, *OsINV4*,* OsMST7*, and *OsMST8*, which contains G‐box sequences. EMSA, electrophoretic mobility shift assay. GST was used as a negative control. (d) *In vitro* EMSA showing that bZIP73^Jap^ could not bind to promoters of *qLTG3‐1* ^*Nip*^, *OsINV4*,* OsMST7*, and *OsMST8*, which contains mutated G‐box (MG) sequences. GST was used as a negative control. (e) Yeast one‐hybrid assay showing that bZIP73^Jap^ activated the expression of p*qLTG3‐1* ^*Nip*^::LacZ, p*O* *sINV4*::LacZ, p*O* *sMST7*::LacZ, and p*O* *sMST8*::LacZ. While, bZIP73^Jap^ could not activate the expression of empty LacZ.

Chromatin immunoprecipitation (ChIP) assays using flowers of bZIP73^Jap^::Flag overexpression lines moreover showed that the anti‐Flag antibody specifically precipitated promoter sequences of the ABA biosynthesis genes *OsNCED3* and *OsNCED5* (Figure 5a), indicating that bZIP73^Jap^ binds to the promoters of these two genes during the reproductive stage. In addition, expression analyses showed that *OsNCED3* and *OsNCED5* were up‐regulated in anthers of 73^Jap^Ri, 71Ri and 73^Ind^OE lines (Figure S6B,D,E), which was consistent with the observed higher ABA levels in anthers of those lines under cold stress conditions (Figure 4a). In contrast, *OsNCED3* and *OsNCED5* genes were down‐regulated in anthers of 71‐73^Jap^OE lines (Figure S6F), resulting in the observed lower ABA levels in anthers of 71‐73^Jap^OE lines under cold stress conditions (Figure 4a).

### bZIP71 enhances bZIP73^Jap^ activity of reducing sugar accumulation in spikelets under cold stress

ABA plays an important role in integrating sugar signaling, sugar metabolism, and sugar distribution (Sharma and Nayyar, 2016). It has been shown that increased ABA levels regulate expression of the tapetum cell wall‐bound invertase gene *OsINV4*, which cleaves sucrose in rice anthers and plays a key role in sugar regulation under cold stress conditions (Oliver *et al*., 2005, 2007). ABA also regulates the monosaccharide transporter genes *OsMST7* and *OsMST8*, which transport glucose and fructose from anther to tapetum and developing pollen grains, and they are critical for pollen development (Mamun *et al*., 2010; Oliver *et al*., 2005, 2007; Sharma and Nayyar, 2016). Yeast one‐hybrid (Y1H) assays showed that the GAL4 activation domain (AD), fusion protein AD‐bZIP73^Jap^ binds to the promoters of *OsINV4*,* OsMST7*, and *OsMST8*, and activates the expression of the LacZ reporter gene (Figure 5e). Furthermore, transient expression assays in rice protoplasts showed that bZIP73^Jap^ *trans*‐activates these promoters and their activities were enhanced by the addition of bZIP71 (Figure 5b). Promoter analyses revealed that the promoters of these genes contain bZIP binding G‐box motifs (Figure S7). To further investigate binding of bZIP73^Jap^ to the promoter regions of these three genes, *in vitro* electrophoretic mobility shift assays (EMSA) were done with recombinant protein GST‐bZIP73^Jap^. Shifted bands were clearly detected when probes containing G‐box elements in the promoter regions of these three genes were incubated with the GST‐bZIP73^Jap^ fusion protein (Figure 5c). Furthermore, we found that bZIP71 enhanced such interactions *in vitro* (Figure 5c). Similarly, ChIP assays showed that bZIP73^Jap^ also specifically precipitated these promoter sequences in flowers of bZIP73^Jap^::Flag overexpression lines (Figure 5a). Thus, our data demonstrated that bZIP73^Jap^ is an upstream transcriptional regulator of *OsINV4*,* OsMST7* and *OsMST8*.

Cold stress during pollen development was shown to result in an accumulation of sucrose in spikelets and depletion of starch in mature pollen grains (Oliver *et al*., 2007). bZIP73^Jap^ can regulate genes encoding a cell wall invertase and monosaccharide transporters, leading to a change of soluble sugar content in anthers under cold stress. Therefore, we examined the soluble sugar content in anthers of transgenic lines and wild‐type ZH11 plants under both normal and natural cold stress conditions, indicating that the abundance of glucose, fructose, and sucrose in 71Ri, 73^Jap^Ri and 73^Ind^OE lines was increased dramatically compared to ZH11 under cold stress (Figure 4b–d). In contrast, levels of glucose, fructose, and sucrose in the 71‐73^Jap^OE lines were significantly decreased compared to ZH11 under cold stress (Figure 4b–d), while all transgenic lines and wild‐type ZH11 had no significant differences in levels of those sugars under warm temperature growth conditions (Figure S5B‐D). The expression levels of *OsINV4*,* OsMST7* and *OsMST8* in flowers of those transgenic lines were well correlated with changes in soluble sugars, which were down‐regulated in the anthers of 73^Jap^Ri, 71Ri and 73^Ind^OE lines (Figure S6B,D,E), but up‐regulated in the anthers of 71‐73^Jap^OE lines (Figure S6F).

### bZIP73^Jap^ interacts with the qLTG3‐1^Nip^ protein and activates its downstream genes

**Figure 6:**
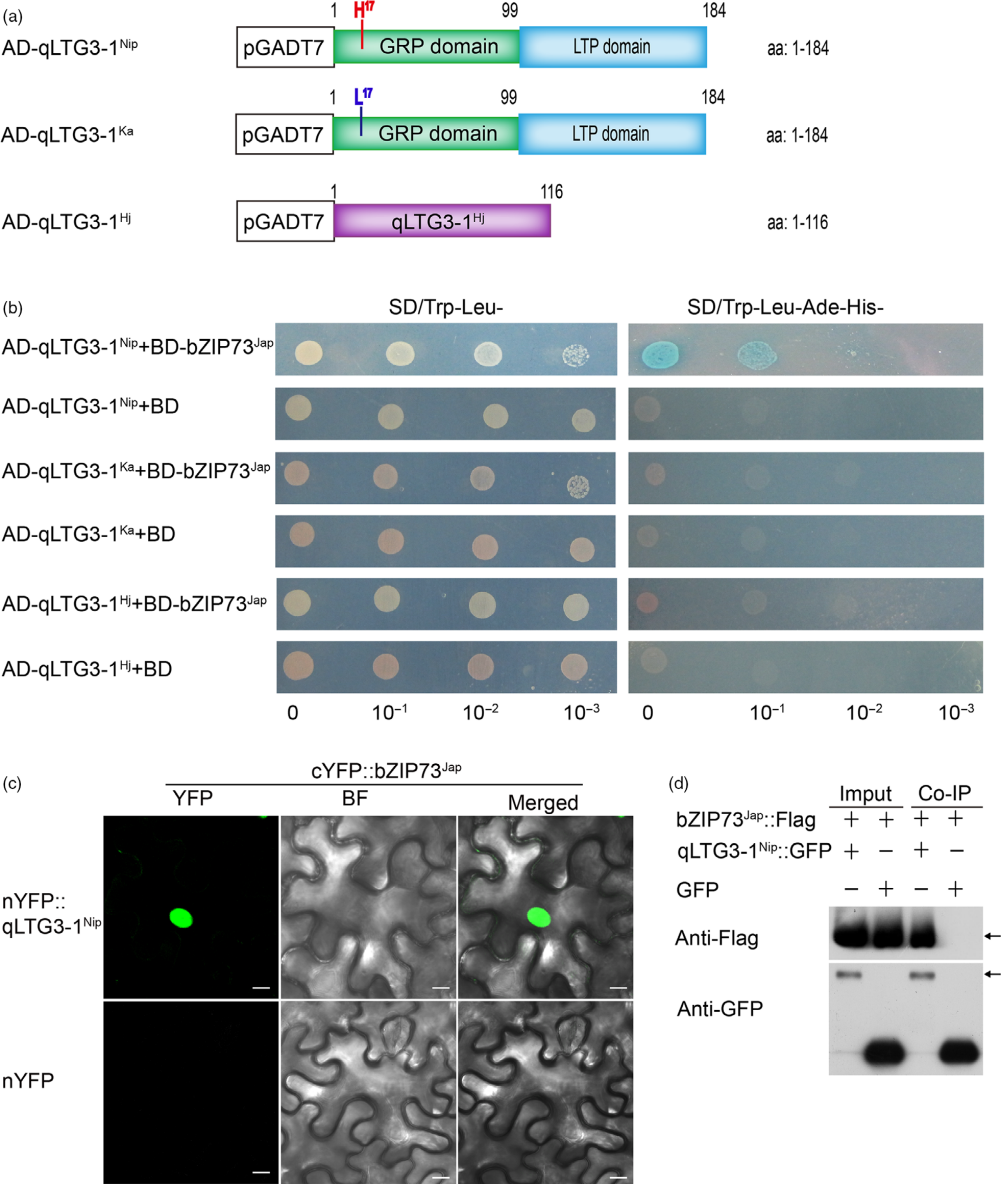
Protein‐protein interactions among bZIP73^Jap^ and qLTG3‐1. (a) Graphic representation of the qLTG3‐1 protein structure. (b) bZIP73^Jap^ interacts with qLTG3‐1^Nip/ka/Hj^ in yeast‐two hybrid (Y2H) assay. *bZIP73* ^*Jap*^ was fused to the GAL4 binding domain (BD) as bait; *qLTG3‐1* ^*Nip*^, *qLTG3‐1* ^*Ka*^, or *qLTG3‐1* ^*Hj*^ was fused to the GAL4 activation domain (AD) as prey, the empty BD as negative control, prey and bait co‐transformated into yeast Y2HGOLD cells. Shown are growth phenotypes of yeast transformants on selective media of SD/Trp‐Leu‐ (left panel) and SD/Trp‐Leu‐His‐Ade‐ (right panel). Ka: Kasalath; ZH11: Zhonghua11; Hj: Hejiang19. (c) bZIP73^Jap^ interacts with qLTG3‐1^Nip^ in a BiFC assay. bZIP73^Jap^ was fused to the C‐terminal region of vernus fluorescent protein, qLTG3‐1^Nip^ was fused to the N‐terminal region of vernus fluorescent protein. Co‐expression of the cYFP‐bZIP73^Jap^ and nYFP‐qLTG3‐1^Nip^ in *N. benthamiana* leaves. Fluorescence observed by confocal microscopy. YFP fluorescence (left), bright field (middle) and bright field overlay images (right) are depicted. Scale bars = 10 μm. (d) Detection of bZIP73^Jap^ and qLTG3‐1^Nip^ interaction by co‐immunoprecipitation analysis. GFP‐tagged bZIP73^Jap^ and Flag‐tagged bZIP73^Jap^ were expressed in *N. benthamiana* leaves for the analysis. Left panel (Input), before precipitation; Right panel (Co‐IP), Anti‐Flag and anti‐GFP antibodies were used to detect Flag and GFP peptides, respectively.

Using a yeast two‐hybrid (Y2H) screen, we previously identified 23 bZIP73^Jap^ interacting proteins (Liu *et al*., 2018a). One of them was qLTG3‐1, which was shown to be involved in rice low‐temperature germinability (Fujino *et al*., 2008). Interestingly, our analysis of the three different *qLTG3‐1* haplotypes: a Nipponbare haplotype (Nip); a Kasalath haplotype (Ka, which is identical to the allele from Italica Livorno); and a Hejiang19 haplotype (Hj, a 71‐bp deletion in the *qLTG3‐1* gene, which is identical to the allele from Hayamasari). To examine interactions between bZIP73^Jap^ and different haplotypes of qLTG3‐1, we fused the full‐length CDS of *bZIP73* ^*Jap*^ to the GAL4 DNA‐binding domain (BD) as bait, while the full‐length CDSs of the three different *qLTG3‐1* alleles (Figures 6a, S8) from Nip, Ka, and Hj were fused to the GAL4 DNA‐activation domain (AD) as prey. In the Y2H assay, only the transformants AD‐qLTG3‐1^Nip^ (aa: 1‐184) + BD‐bZIP73^Jap^ could grow on the quadruple dropout medium SD/Trp‐Leu‐His‐Ade‐ plates, while the transformants AD‐qLTG3‐1^Ka^ (aa: 1‐184) + BD‐bZIP73^Jap^ and AD‐qLTG3‐1^Hj^ (aa: 1‐116) + BD‐bZIP73^Jap^ could not grow on quadruple dropout medium (Figure 6b). These results demonstrated that bZIP73^Jap^ interacts directly with qLTG3‐1^Nip^, but not qLTG3‐1^Ka^ or qLTG3‐1^Hj^. The interaction between bZIP73^Jap^ and qLTG3‐1^Nip^ was further confirmed *in vivo* using bimolecular fluorescence complementation (BiFC) assays and co‐immunoprecipitation (Co‐IP) experiments in *Nicotiana benthamiana* by agroinfiltration (Figure 6c,d).

Y1H, EMSA and ChIP assays showed that the bZIP73^Jap^ protein specifically binds to the promoter sequence of *qLTG3‐1* ^*Nip*^ (Figure 5a,c–e). Transient expression assays in rice protoplasts also showed that bZIP73^Jap^ *trans*‐activates the *qLTG3‐1* promoter and this activity was enhanced by the addition of bZIP71 (*P *< 0.01, one way anova test) (Figure 5b). Correspondingly, the expression of *qLTG3‐1* ^*Nip*^ was down‐regulated in 73^Jap^Ri lines (Figure S6E). Furthermore, bZIP73^Jap^ activates *qLTG3‐1* ^*Nip*^, *OsMST7*,* OsMST8* and *OsINV4*, and addition of the qLTG3‐1^Nip^ protein enhances this activation in transient expression assays of rice protoplasts (*P *< 0.01, one way anova test; Figure 5b). Thus, our data demonstrated that bZIP73^Jap^ interacts with qLTG3‐1^Nip^, and this protein‐protein interaction enhances bZIP73^Jap^ activation of downstream target gene expression.

### *qLTG3‐1* ^*Nip*^ overexpression transgenic lines improve cold tolerance

**Figure 7:**
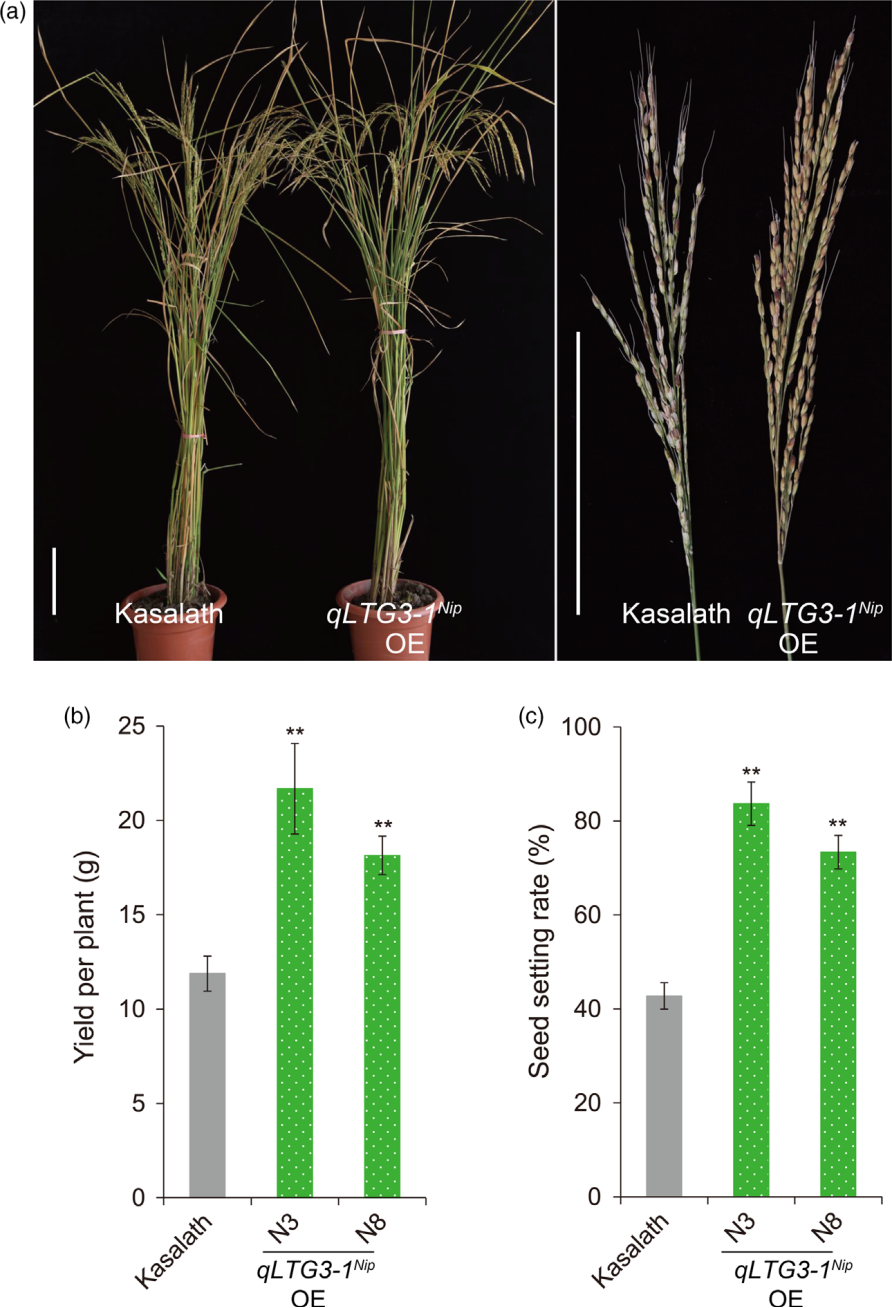
Cold tolerance phenotypes and agronomic traits of *qLTG3‐1* ^*Nip*^ overexpression transgenic lines experiencing natural cold stress conditions during the reproductive stage grown in the autumn of 2012 in Beijing (heading date: September 12). (a) Phenotypes of *qLTG3‐1* ^*Nip*^ overexpression (*qLTG3‐1* ^*Nip*^ OE) lines and Kasalath wild‐type control plants, (b) Yield per plant, (c) Seed‐setting rate. Scale bars = 15 cm. Three biological replicates (*n* = 30 each) were used per line. Error bar, standard deviation. **P < 0.01, two‐tailed *t*‐test in comparison to ZH11.

**Figure 8:**
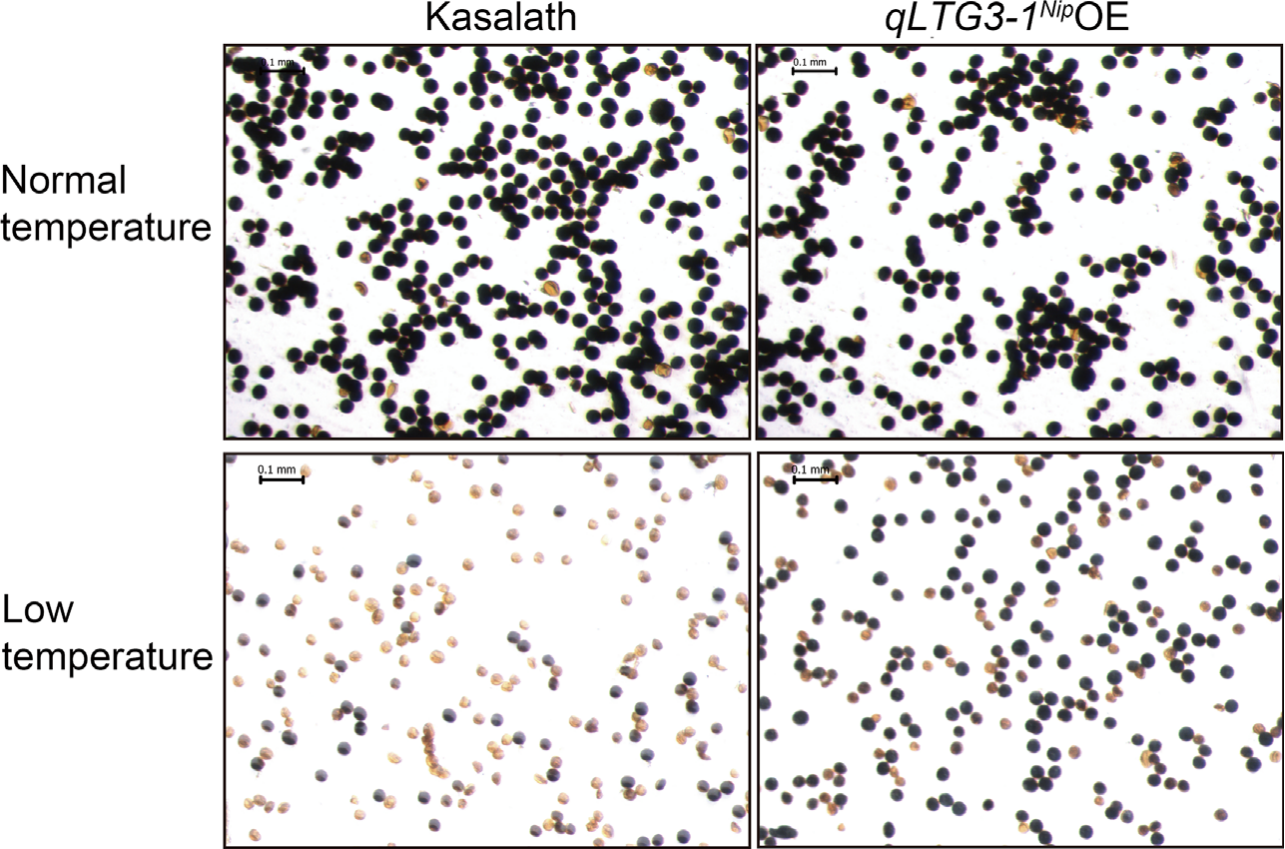
KI/I_2_ staining phenotype of pollen grains from *qLTG3‐1* ^*Nip*^ overexpression lines and Kasalath control plants under natural cold stress conditions as a measure of the effect of cold treatment on pollen viability. Shown are staining phenotypes of pollen grains of plants grown under normal warm temperature conditions in Beijing (heading date: August 13), and natural cold stress conditions with average terrestrial temperatures below 20 °C (Figure S3; heading date: September 12). Scale bars = 0.1 mm. Microscopic observation shows an increased amount of starch (dark blue color) in pollen grains from *qLTG3‐1* ^*Nip*^ overexpression lines compared to pollen grains from Kasalath control plants after experiencing natural cold stress conditions.

It was previously shown that *qLTG3‐1* is specifically expressed in panicles and embryos of germinating seeds (Fujino and Matsuda, 2010; Fujino *et al*., 2008). Our expression analyses revealed that *qLTG3‐1* ^*Nip*^ is predominantly expressed in panicles at the EB and flowering stages (Figure S1) and up‐regulated by cold stress (Figure S9). These results suggested that *qLTG3‐1* ^*Ni*p^ might play an important role in cold stress tolerance during the reproductive stage. To further characterize *qLTG3‐1* function, three different alleles of *qLTG3‐1* were introduced into the *indica‐*type cultivar Kasalath via *Agrobacterium*‐mediated transformation. Analyses of the transgenic plants harboring *qLTG3‐1* ^*Nip*^ demonstrated that the seed‐setting rate of *qLTG3‐1* ^*Nip*^ overexpression (OE) lines improved by 30.6%–41%, and yield per plant improved by 52.7%–82.5% compared to wild‐type Kasalath under natural cold stress conditions (Figure 7, Table S4). Seed‐setting rate and yield per plant were positively correlated with expression levels of *qLTG3‐1* ^*Nip*^ (Figure 7, Figure S10). Moreover, *qLTG3‐1* ^*Nip*^ OE lines reduced soluble sugar (sucrose, glucose and fructose) accumulation in flowers (Figure S11), but compared to wild‐type Kasalath, increased pollen fertility under natural cold stress conditions (Figure 8). Similar results were obtained with another batch of field trial under natural cold stress conditions (Table S5). Surprisingly, both *qLTG3‐1* ^*Ka*^ OE and *qLTG3‐1* ^*Hj*^ OE lines showed no differences compared to Kasalath under natural cold stress conditions (Tables S4 and S5). All agronomic traits of transgenic lines harboring the three different alleles of *qLTG3‐1* were not different compared to Kasalath under normal growth conditions (Table S6). These results indicated that *qLTG3‐1* ^*Nip*^ OE lines significantly improve rice tolerance to cold stress during the reproductive stage.

## Discussion

ABA plays a critical role in mediating cold stress responses in plants (Mittler and Blumwald, 2015). Rice mutants deficient in carotenoid biosynthesis genes, the mutants deficient in ABA biosynthetic precursors, and plants overexpressing the ABA‐catabolic gene *OsABA8ox1* have reduced ABA levels and are more tolerant to cold stress than wild‐type plants at the seedling stage (Du *et al*., 2013; Mega *et al*., 2015). It was also shown that cold‐induced accumulation of ABA was higher in anthers of cold‐sensitive cultivars than cold‐tolerant cultivars (Oliver *et al*., 2007). Overexpression of the wheat ABA catabolic gene *TaABA8′OH1* resulted in reduced ABA levels in anthers and improved cold tolerance of rice (Ji *et al*., 2011). Our work revealed that 71‐73^Jap^OE transgenic lines had lower ABA levels and were more tolerant to cold stress at the seedling stage (Liu *et al*., 2018a), and as shown here, also at the reproductive stage, while 71Ri, 73^Jap^Ri, 73^Ind^OE lines had higher ABA levels and were more sensitive to cold stress than wild‐type plants, indicating that a reduction of ABA levels improved cold tolerance both at the seedling and reproductive stage in rice.

ABA also plays an important role in regulating sugar signaling and enhances the ability of plant tissues to respond to subsequent sugar signals and repression of *OsINV4* and *OsMST8* (Rook *et al*., 2001; Wang *et al*., 2015). The invertase OsINV4 catalyzes the hydrolysis of sucrose to glucose and fructose, which plays a critical role in sugar partitioning and regulating source‐sink interactions under cold stress conditions (Mamun *et al*., 2006; Oliver *et al*., 2005, 2007; Tymowskalalanne and Kreis, 1998). Under cold stress, reduced expression of the monosaccharide transporter gene *OsMST8* and the invertase gene *OsINV4* in chilling sensitive rice cultivars results in perturbed carbohydrate metabolism and reduced sugar transport to the tapetum and developing pollen grains, thus causing pollen abortion and lower pollen number (Oliver *et al*., 2005, 2007; Sakata *et al*., 2014). bZIP73^Jap^ *trans*‐activates the expression of *OsINV4*,* OsMST7*, and *OsMST8*, and bZIP71 enhances this activity. High expression level of *bZIP71* and *bZIP73* ^*Jap*^ in 71‐73^Jap^OE lines leads to up‐regulation of *OsINV4*,* OsMST7* and *OsMST8*. Meanwhile, 71‐73^Jap^OE lines have lower ABA levels in anthers during cold stress than wild‐type controls, which attenuates repression of *OsINV4*,* OsMST8*, resulting in enough soluble sugars transported to pollen in 71‐73^Jap^OE lines, which improves pollen grain fertility, seed‐setting rate, and overall yield under cold stress conditions. In contrast, expression levels of *bZIP73* ^*Jap*^ and *bZIP71* in 71Ri, 73^Jap^Ri, and 73^Ind^OE lines are lower than in wild‐type ZH11, leading to down‐regulated expression of *OsINV4*,* OsMST7* and *OsMST8*. Because cold stress causes ABA accumulation in anthers of those lines, increased ABA levels would feedback inhibit the expression of *OsINV4* and *OsMST8*, leading to reduced soluble sugars transported to the pollen grains. Therefore, sugars accumulate in anthers instead of pollens and pollen grains become sterile, leading to low seed‐setting rate and reduced yield under cold stress conditions.

Both bZIP73 and bZIP71 belong to bZIP transcription factor group S (Liu *et al*., 2014). In *Arabidopsis*, Group S members of bZIP transcription factors such as AtbZIP1, AtbZIP2/GBF5, AtbZIP11/ATB2, AtbZIP44, and AtbZIP53 play important roles in sugar signaling (Kang *et al*., 2010; Ma *et al*., 2011; Wiese *et al*., 2004). bZIP71 enhances bZIP73^Jap^ in regulating soluble sugar allocation between source (anthers) and sink (pollen) under cold stress, suggesting that *bZIP73* ^*Jap*^ has a conserved function in Group S and plays a role in the cross‐talk of ABA and sugar signaling.

Interestingly, 73^Ind^OE lines are as cold sensitive as 73^Jap^Ri lines with a loss‐of‐function phenotype. A possible explanation for this is that the 73^Ind^OE lines were made by overexpressing *bZIP73* ^*Ind*^ in a *japonica* cultivar, Zhonghua 11, which contains an endogenous allele of *bZIP73* ^*Jap*^. Since both bZIP73^Jap^ and bZIP73^Ind^ can form heterodimer with bZIP71 *in vitro* and *in vivo*, there is competition between bZIP73^Ind^ and bZIP73^Jap^ for interaction with bZIP71, which reduces the endogenous amount of productive bZIP73^Jap^‐bZIP71 complexes in transgenic lines and thus might have a dominant‐negative effect (Liu *et al*., 2018a). This suggests that reduction of endogenous bZIP73^Jap^‐bZIP71 complexes leads to the down‐regulation of *qLTG3‐1* ^*Nip*^, monosaccharide transporter genes, and cell wall invertase genes (Figure S6), and thus reduced soluble sugars transport from anthers to pollens and reduced pollen grain number (Figure 3a,c), seed‐setting rate, and grain yield in 73^Ind^OE lines (Figure 2a–c). The phenotype of 73^Ind^OE lines at the reproductive stage was similar to that at the seedling stage (Liu *et al*., 2018a).

The qLTG3‐1 protein has two conserved domains, a glycine‐rich protein (GRP) motif and a lipid transfer protein (LTP) motif (Fujino *et al*., 2008). *qLTG3‐1* and its orthologs are conserved among rice, *Brachypodium*, sorghum, maize, and dicots such as Arabidopsis, and orthologous genes have a conserved function during seed germination (Fujino *et al*., 2014; Xu *et al*., 2011). GRP domains are functionally conserved in plants, and many genes coding for those domains are involved in cold signaling. *AtGRP2*,* AtGRP4*,* AtGRP7* and *AtRZ‐1b* in *Arabidopsis* exhibit RNA chaperone activity during the cold adaptation process (Kim *et al*., 2007, 2010b; Kwak *et al*., 2011). In rice, plants overexpressing *OsGRP1*,* OsGRP4*, and *OsGRP6* were more tolerant to low temperature stress (Kim *et al*., 2010a). In *Nicotiana tabacum*,* NtGRP1*,* NtGRP‐1a* and *NtGRP3* transcripts were up‐regulated after cold treatment (Chen *et al*., 2010; Czolpinska and Rurek, 2018). It was previously shown that the Italica Livorno allele of *qLTG3‐1*, which is identical to the Kasalath allele, *qLTG3‐1* ^*Ka*^, has a much stronger activity than the Nipponbare allele of *qLTG3‐1*,* qLTG3‐1* ^*Nip*^, in conferring low‐temperature germinability and pre‐harvest sprouting resistance (Fujino *et al*., 2008; Hori *et al*., 2010; Iwata and Fujino, 2010). In our study, however, only the Nipponbare allele *qLTG3‐1* ^*Nip*^ conferred cold stress tolerance in rice at the reproductive stage, while the Kasalath and Hejiang 19 alleles *qLTG3‐1* ^*Ka*^ and *qLTG3‐1* ^*Hj*^ were non‐functional in improving cold stress tolerance at that stage. Compared to the Kasalath allele *qLTG3‐1* ^*Ka*^, *qLTG3‐1* ^*Nip*^ has only one nucleotide change leading to a nonsynonymous substitution in the 17^th^ amino acid, resulting in the novel function of cold tolerance at the reproductive stage. Uncovering in future work the molecular mechanism of how this single amino‐acid change results in a novel functional allele that improves cold stress tolerance during the reproductive stage will be extremely interesting for understanding mechanisms of cold stress tolerance in rice. One possibility is that the amino acid change affects the subcellular localization of qLTG3‐1. In agreement with this, the subcellular localization of fluorescent signals of the qLTG3‐1^Ka^::GFP fusion protein was stronger than that of the qLTG3‐1^Nip^::GFP fusion, (Figure S12), possibly because the amino acid substitution is within the presumed N‐terminal signal sequence of the protein.

Although qLTG3‐1^Nip^ cannot activate *OsMST7*,* OsMST8* or *OsINV4* expression (Figure 5b), *qLTG3‐1* ^*Nip*^ OE lines nonetheless have a reduced level of soluble sugars (sucrose, glucose and fructose) in flowers under natural cold stress conditions (Figure S11). A possible explanation for this is that the physical interaction of qLTG3‐1^Nip^ with bZIP73^Jap^ enhances the expression of *OsMST7*,* OsMST8* and *OsINV4*, thus causing *qLTG3‐1* ^*Nip*^ OE lines to transport more soluble sugars than wild‐type plants from the source (flowers) to the sink (pollen), leading to reduced soluble sugar accumulation in flowers (Figure S11), improved pollen fertility (Figure 8), seed‐setting rates, and yield per plant (Figure 7).

Previous ChIP‐qPCR analysis showed that bZIP73^Jap^ binds to the promoter regions of peroxidase precursors (POXs, *LOC_Os01g22249*,* LOC_Os03g02920*,* LOC_Os03g32050* and *LOC_Os04g59210*) in seedlings of bZIP73^Jap^::Flag OE lines, and up‐regulated their expression in *bZIP71‐bZIP73* ^*Jap*^ OE (71‐73^Jap^OE) lines (Liu *et al*., 2018a). However, the factor cannot bind to the promoters of *qLTG3‐1* ^*Nip*^, *OsINV4*,* OsMST7*, and *OsMST8* at the seedling stage (Figure S13B), and there is no significant difference in expression levels of those genes between 71‐73^Jap^OE lines and wild‐type ZH11 (Figure S13D). In contrast, ChIP‐qPCR showed that bZIP73^Jap^ binds to the promoters of *qLTG3‐1* ^*Nip*^, *OsMST7*,* OsMST8*, and *OsINV4*, in the panicles of bZIP73^Jap^::Flag OE lines (Figure 5a), but not to the promoters of the four POX genes (Figure S13A), nor does it change their expression in panicles of the 71‐73^Jap^OE lines (Figure S13C). *qLTG3‐1* ^*Nip*^ is predominantly expressed in panicle at EB and flowering stages (Figure S1), while *OsMST7 OsMST7*,* OsMST8* and *OsINV4* were expressed predominantly in pollen, anther wall, or tapetum from the YM until the pollen maturity stage (Oliver *et al*., 2005, 2007). A possible explanation for these tissue specific expression differences is that the chromatin structure at loci of *qLTG3‐1* ^*Nip*^, *OsMST7*,* OsMST8*, and *OsINV4* might be open and accessible for bZIP73^Jap^ or bZIP71:bZIP73^Jap^ at the reproductive stage, while it might be closed and inaccessible for those factors at the seedling stage. This means that a combination of chromatin accessibility and possible interactions with different binding partners allows transcription factors such as bZIP73^Jap^ or bZIP71:bZIP73^Jap^ to regulate different target genes at different development stages. On the other hand, expression levels of *bZIP71* and *bZIP73* ^*Jap*^ in response to cold were also different in vegetative seedling and at the reproductive stage. In shoots of two‐week seedlings, the expression of *bZIP71* was much higher than that of *bZIP73* ^*Jap*^ (Figure S14), while expression levels of those two genes were similar in panicles at the reproductive stage (Figure S14). Moreover, the expression of *bZIP73* ^*Jap*^ did not change during cold exposure in shoots at the seedling stage (Liu *et al*., 2018a), while it was cold induced in flag leaves and panicles at the reproductive stage (Figure 1). At the same time, expression levels of *bZIP71* did not change in response to cold conditions both at the seedling (Liu *et al*., 2014) and reproductive stages (Figure S2). Therefore, the abundance of bZIP71:bZIP73^Jap^ heterodimers is most likely different at the seedling and reproductive stages, thus activation of downstream genes is different. Furthermore, because bZIP73^Jap^ binds to the promoter of *qLTG3‐1* ^*Nip*^, which is preferentially expressed in panicles at EB and flowering stages (Figure S1), and also physically interacts with qLTG3‐1^Nip^ (Figure 6), there might be a feedback regulation of *qLTG3‐1* ^*Nip*^ on the transcriptional activity of bZIP73^Jap^ as follows: bZIP73^Jap^ upregulates *qLTG3‐1* ^*Nip*^ at the rice reproductive stage, thus promoting a physical interaction between qLTG3‐1^Nip^ and bZIP73^Jap^, which in turn enhances expression of reproductive stage specific bZIP73^Jap^ target genes such as *qLTG3‐1* ^*Nip*^, *OsMST7*,* OsMST8* and *OsINV4* (Figure 5b). Alternatively, interaction of qLTG3‐1^Nip^ and bZIP73^Jap^ may sequester the bZIP71‐bZIP73^Jap^ heterodimer mainly to those four target genes, thus preventing the bZIP71‐bZIP73^Jap^ heterodimer from activating the expression of POX genes at the reproductive stage. Moreover, the effect of transcription factors on their target genes usually varies depending on particular developmental or cellular contexts (Lu *et al*., 2013; Spitz and Furlong, 2012), and genes associated with bZIP73^Jap^ binding sites might be differently expressed at the seedling and reproductive stages. This would suggest a developmental or cellular context dependent regulation of bZIP73^Jap^.

**Figure 9:**
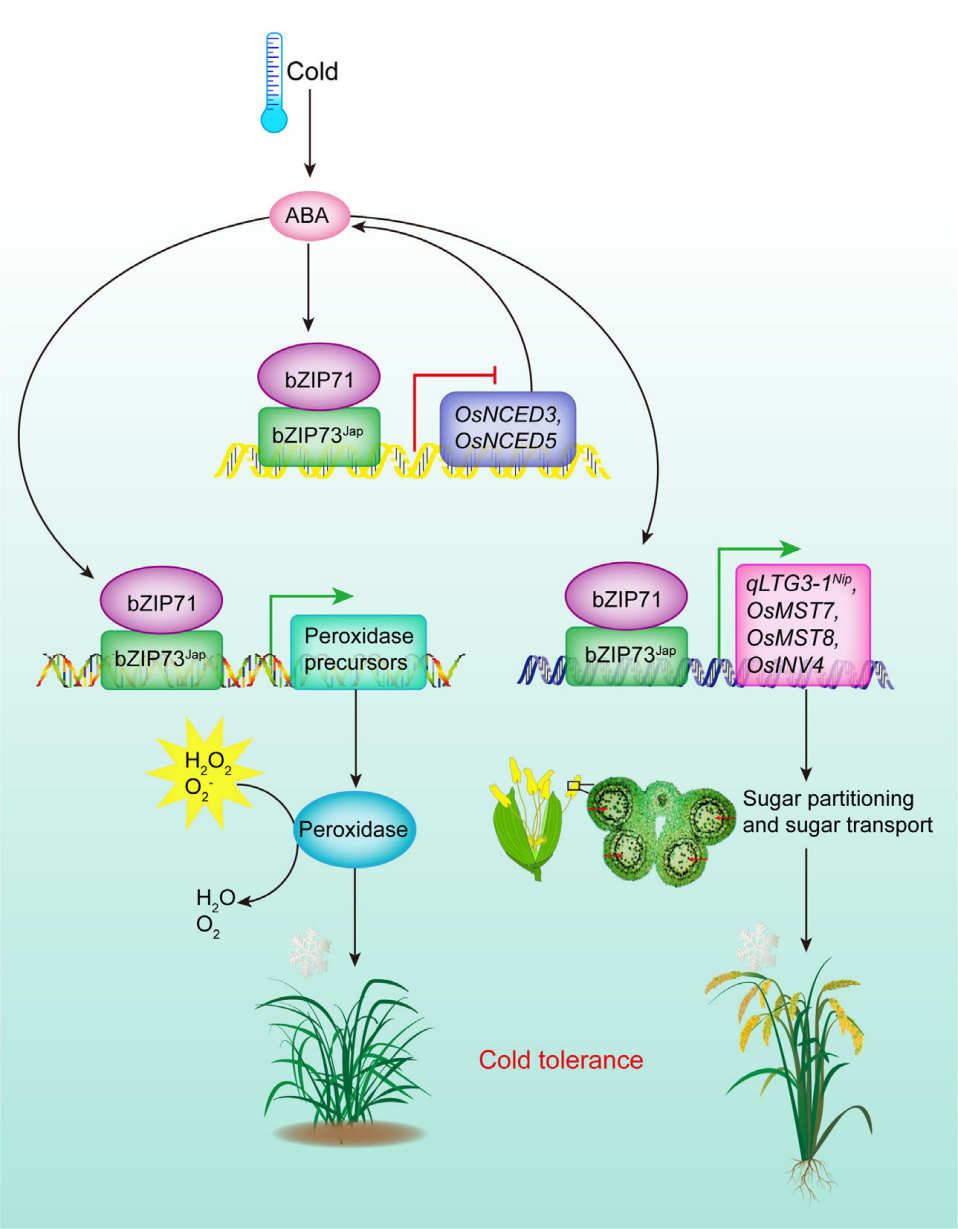
Proposed model for the molecular mechanisms of bZIP73^Jap^‐mediated cold tolerance in rice plants at both the seedling and the reproductive stage.

Based on our results, we propose the following model for rice cold tolerance at the reproductive stage: when rice encounters cold stress, bZIP73^Jap^ is induced which recruits bZIP71 to form heterodimers, leading to repressed ABA biosynthesis and activated peroxidase expression, and ultimately improved cold stress tolerance at the seedling stage (Liu *et al*., 2018a). During the reproductive stage, bZIP71:bZIP73^Jap^ forms heterodimers and activates transcription of *qLTG3‐1* ^*Nip*^, monosaccharide transporter genes, and cell wall invertase genes, which enhances soluble sugar transport from anthers to pollen, thus improving the rice seed‐setting rate under cold stress conditions (Figure 9).

## Materials and methods

### Plant materials and growth conditions

The *Japonica* cultivar (cv.) Zhonghua 11 (ZH11) was used to generate *bZIP71* (*LOC_Os09g13570*), *bZIP73* ^*Jap*^ (*LOC_Os09g29820*), *bZIP73* ^*Ind*^ overexpression (71OE, 73^Jap^OE, 73^Ind^OE), *bZIP71* and *bZIP73* ^*Jap*^ co‐expression (71‐73^Jap^OE), and *bZIP71*,* bZIP73* ^*Jap*^ RNAi transgenic lines (71Ri, 73^Jap^Ri). The *Indica* type cv. Kasalath was used to generate *qLTG3‐1* ^*Nip*^, *qLTG3‐1* ^*Ka*^, and *qLTG3‐1* ^*Hj*^ overexpression transgenic lines. Rice plants were grown in a paddy field under natural conditions in Beijing (116.4°E/39.9°N).

### Expression analyses with real‐time PCR

For analysis of gene expression patterns in response to cold stress during the reproductive stage, anther developmental stages were determined based on the AD method [AD: the distance in cm between the nodes of the flag leaf and the penultimate leaf (Oliver *et al*., 2005)]. The YM stage was AD 0 to +2.5 cm; the EB stage was AD +6 to +8 cm; and the flowering stage was AD: +16 to 18 cm. Cold treatment was carried out in a controlled growth chamber (14 h light/10 h dark) for 1 day at a constant temperature of 12 °C, and the panicles and flag leaves of ZH11 were collected 0, 0.25, 0.5, 1, 3, 6, 12, and 24 h after cold treatment. For tissue expression pattern analysis, different ZH11 tissues including shoots (two‐week‐old), roots (two‐week‐old), stems (YM stage), flag leaves (YM stage), panicles (YM and EB stages), and flowers (1 day before flowering) were used for RNA extraction and quantitative RT‐PCR.

Total RNA was prepared from rice tissues using the Trizol reagent (Invitrogen, Carlsbad, CA, USA) according to the manufacturer's protocol. Total RNA was reverse transcribed to cDNA using the ReverTra Ace qPCR RT Master Mix with gDNA remover Kit (Toyobo, Osaka, Japan) according to the manufacturer's instruction. cDNA templates were used for real‐time PCR analysis. Real‐time PCR was done using the SYBR^®^ Premix Ex TaqTM Kit (TaKaRa, Kusatsu, Japan) protocol in an ABI PRISM 7900HT (Applied Biosystems, Foster City, CA, USA). The rice *Ubiquitin* gene (*LOC_Os03g13170*) was used as internal control. Primers for real‐time PCR are listed in Table S7. Data analysis was conducted as described by Pfaffl (Pfaffl, 2001).

### Vector construction and plant transformation

To avoid negative agronomic traits in transformed plants caused by fusion tags of binary expression vectors, overexpression vectors were constructed without fusion tags. Three different alleles of *qLTG3‐1* (*LOC_Os03g01320*) coding sequences (CDS) were amplified by RT‐PCR from Nipponbare, Kasalath, and Hejiang19, and subsequently cloned into the binary vector pCambia1390‐Ubiquitin to generate the pCAMBIA1390‐Ubiquitin‐ *qLTG3‐1* ^*Nip/Ka/Hj*^ overexpression constructs. The CDS of *bZIP71*/*bZIP73* ^*Jap/Ind*^ was cloned into pENTER‐TOPO vectors (Invitrogen, Carlsbad, CA, USA), then they were cloned into the binary vector pH2GW7 (Karimi *et al*., 2002) (Invitrogen) using the LR reaction of the pENTER‐TOPO vectors, to generate the pH2GW7‐*bZIP71/bZIP73* ^*Jap/Ind*^ overexpression vectors. All binary constructs were introduced into *Agrobacterium tumefaciens* strain AGL1 and subsequently transformed into the *Indica* type cultivar Kasalath. Primer sequences for vector construction are listed in Table S7.

### Stress tolerance assays

For field test of cold resistance at the reproductive stage, transgenic lines were planted in the temperate zone of Beijing (39.9°N) in Northern China, where temperatures in late August and early September are relatively low, with average daily temperatures usually below 20 °C. Exposure of rice to temperatures below 20 °C at the booting stage causes pollen sterility or fertilization failure. Different planting date experiments using transgenic lines were conducted in Beijing every fifteen days from May 10th to July 9th, which ensured cold temperature would hit at the rice booting stage in late August or early September. Because the constitutive *bZIP71* overexpression lines (71OE) flowered 7 days later than wild‐type ZH11 (Liu *et al*., 2014), we sowed 71OE lines 7 days earlier than wild‐type and other transgenic lines to ensure that the heading date of each batch was the same. Thirty plants of each line were used for each plot, and the experiments were replicated at least three times. Agronomic traits including plant height, tiller number, seed‐setting rate, and yield per plant were statistically analyzed.

### Microscopy

Starch staining assays of pollen grains (Nelson, 1968) were performed just one day before flowering. For the morphological observations of anthers, samples were fixed in a Formalin‐Aceto‐Alcohol (FAA) solution. To determine pollen fertility, fixed anthers were ground to release pollen grains, stained with I_2_‐KI solution, then observed microscopically and photographs taken with an Olympus SEX16. Blue stained pollen grains were counted as a measure to determine pollen fertility. Pollen or male sterility is defined as an absence of or presence of non‐functional pollen grains in plants, or as the inability of plants to produce or release functional pollen grains. Immature pollen is sterile. One way avova test, *P*‐value in Table S8.

### ABA quantification

Quantification of endogenous ABA was performed as described previously (Li *et al*., 2011). Rice flowers (approximately 3 g of fresh weight) of transgenic lines and wild‐type plants were harvested one day before flowering both under normal (heading at August 17, 2012, Beijing) and natural cold conditions (heading at September 13, 2012, Beijing). Each series of experiments were performed using biological triplicates.

### Sugar measurements

Samples were harvested one day before flowering both under normal (heading on August 17, 2012, Beijing) and natural cold conditions (heading on September 13, 2012, Beijing). Anthers were ground in liquid nitrogen to a fine powder, and 10 replicates for each line were used. 0.7 mL of 80% ethanol was added to each sample and immediately incubated at 80 °C for 60 min. After cooling, samples were centrifuged at 4 °C and 16 873 ***g*** for 5 min. The supernatant was transferred to a new Eppendorf tube, and the remaining pellet was washed twice with 0.7 mL of 80% ethanol. The supernatant was evaporated in a speed vacuum dryer at 50 °C for around 60–90 min and the residue resuspended in 200–250 μL of water and used directly for measurements. The content of glucose, fructose, and sucrose were measured using enzyme‐based methods as described previously (Spackman and Cobb, 2002). One way avova test, *P*‐value in Table S8.

### EMSA

The CDS of *bZIP71* and *bZIP73* ^*Jap*^ were amplified and cloned into the pGEX4T‐3 vector to generate GST fusion proteins, and were purified as previously reported (Liu *et al*., 2018a). For promoter binding experiments, complementary single‐stranded oligonucleotides derived from 70 bp of the G‐box region of downstream gene (*qLTG3‐1* ^*Nip*^, *OsMST7*,* OsMST8* and *OsINV4*) promoters were synthesized as DNA probes. To obtain double stranded G‐Box motif‐containing fragments, two complementary oligonucleotides were mixed in a water bath at 95 °C for 5 min and cooled to room temperature for annealing. A mutated G‐box sequence was amplified from wild type using mutated primers and was used as negative control. EMSA was performed using a chemiluminescent EMSA kit (Thermo, Massachusetts, USA). Probe sequences are shown in Table S7.

### ChIP‐qPCR

ChIP assays were performed as described previously, with some modifications (Fiil *et al*., 2008). Briefly, about 2 g of flowers from T3 homozygous lines of bZIP73^Jap^::Flag, bZIP73^Ind^::Flag, or bZIP71::Flag transgenic lines in the ZH11 background were cross‐linked in 1% formaldehyde under a vacuum, and cross‐linking was stopped with 0.125 m glycine. Samples were ground to a powder in liquid nitrogen, and nuclei were isolated. An antibody against bZIP fused Flag tag was used at a 1:5000 dilution to immuno‐precipitate protein‐DNA complexes. Immunoprecipitation was performed with anti‐Flag antibody (anti‐Flag) or without antibody (No Ab) as a negative control. Fragments consisting of a 1000 bp sequence upstream of the ATG were chosen as the promoter (Figure S6). This promoter fragment contained G‐box element regions used for designing primers (Table S7). The *Ubiquitin* gene was used as a negative control. The amount of precipitated DNA was calculated relative to the total input of chromatin and expressed as a percentage of the total according to the formula: % input = 2^▵*C*t ^× 100%, where ▵*C*t = *C*t (input) − *C*t (IP) and *C*t is the mean threshold cycle of the corresponding PCR reaction (Chernukhin *et al*., 2011). Three independent biological replicates were used.

### Transient expression in rice protoplasts

For determination of subcellular localization, the full‐length CDSs of *qLTG3‐1* ^*Nip/Ka*^ were inserted into pCambia2300‐35S‐GFP, creating *qLTG3‐1* ^*Nip*^::GFP and *qLTG3‐1* ^*Ka*^::GFP fusion vectors. Plasmid DNA was prepared using the Plasmid Midi Kits (Qiagen, Germany) according to the manufacturer's instructions. *qLTG3‐1* ^*Nip/Ka*^::GFP fusion vectors were transformed into rice (ZH11) leaf protoplasts using the polyethylene glycol method (Zhang *et al*., 2011). After 12 h of incubation in the dark at 28 °C, transient expression of green fluorescent protein (GFP) fluorescence was recorded using a confocal laser‐scanning microscope (Carl Zeiss, LSM510, Germany).

For the Dual‐Luciferase Assay, promoters of *qLTG3‐1* ^*Nip*^, *OsMST7* (*LOC_Os01g38680*), *OsMST8* (*LOC_Os01g58670*), and *OsINV4* (*LOC_Os04g33720*) (~2000 bp upstream of ATG) were amplified and cloned into the pGreenII 0800‐LUC vector containing the firefly luciferase (fLUC) gene and the *Renilla* LUC gene (rLUC) as reporters (Hellens *et al*., 2005), while the CDS of *bZIP71*,* bZIP73* ^*Jap/Ind*^ and *qLTG3‐1* ^*Nip*^ were cloned into the pCambia1300‐Flag vector as effectors. Empty pCambia1300‐Flag vector was used as negative control for the effector. Rice (ZH11) shoot protoplasts were prepared and transfected and followed by a 12 h incubation to allow transient expression (Zhang *et al*., 2011). Protoplasts were collected by centrifugation at 450 *g* for 3 min and immediately utilized for luciferase assays. Luciferase activity was quantified using a dual‐luciferase reporter assay kit (Promega, E1910, Madison, WI, USA). Five independent transformations for each sample were performed, and the relative luciferase activity was calculated as the ratio of fLUC to rLUC (fLUC/rLUC).

### Yeast assays

To prepare constructs for the yeast one‐hybrid (Y1H) assay, the promoter region of *qLTG3‐1* ^*Nip*^, *OsMST7*,* OsMST8* and *OsINV4* (2 kb upstream of ATG) was amplified and ligated into the *Eco*RI‐*Sal*I sites of the pLacZi2μ vector (Lin *et al*., 2007) to generate p*qLTG3‐1* ^*Nip*^ */*p*OsMST7/*p*OsMST8*/p*OsINV4::*LacZ. To generate AD‐bZIP73^Jap^, the full‐length CDS of *OsbZIP73* ^*Jap*^ was ligated into the *Eco*RI‐*Xho*I sites of the pJG4‐5 vector (Clontech). The Y1H assay was performed according to the Yeast Protocols Handbook (Clontech). Briefly, the AD fusion constructs were co‐transformed with various LacZ reporter plasmids into yeast strain EGY48. Transformants were grown on SD/Trp‐Ura‐ dropout plates containing 5‐bromo‐4‐chloro‐3‐indolyl‐ß‐D‐galactopyranoside (X‐Gal) for blue color development.

Yeast two‐hybrid (Y2H) assays were conducted using the Matchmaker Two‐Hybrid System 3 (Clontech). The activation domain (AD) vector pGADT7, the DNA‐binding domain (BD) vector pGBDT7, and the yeast strain Y2HGold were utilized. The full‐length CDSs of *qLTG3‐1* ^*Nip*^ (aa: 1‐184), *qLTG3‐1* ^*Ka*^ (aa: 1‐184), or *qLTG3‐1* ^*Hj*^ (aa: 1‐116) were cloned respectively into the vector pGADT7 as prey, and the full‐length CDS of *bZIP73* ^*Jap*^ was cloned into the vector pGBDT7 as bait. The amplification primers used are shown in Table S7. The prey and bait vectors were co‐transformed into Y2HGold cells and the transformants were identified according to the manufacturer's instructions.

### BiFC and Co‐IP

To generate BiFC constructs, the CDSs of *bZIP73* ^*Jap*^ and *qLTG3‐1* ^*Nip*^ were PCR amplified and ligated into VYCE(R) and VYNE(R) (Waadt *et al*., 2008), resulting in bZIP73^Jap^::cYFP and qLTG3‐1^Nip^::nYFP, respectively. Empty vectors of BiFC constructs were used as negative controls. To generate Co‐IP constructs, the CDSs of *bZIP73* ^*Jap*^ and *qLTG3‐1* ^*Nip*^ were PCR amplified and ligated into pCambia1300‐Flag and pCambia2300‐35S‐GFP, resulting in bZIP73^Jap^::Flag and qLTG3‐1^Nip^::GFP, respectively. Primers used for generating fusion constructs are listed in Table S7. All the constructs were transformed into the *Agrobacterium tumefaciens* GV3101 strain and used for BiFC and Co‐IP experiments.

*Agrobacterium* solutions were adjusted to OD_600 _= 0.8 and then equally mixed before Agroinfiltration. An *Agrobacterium* strain carrying the viral suppressor p19 was used to suppress gene silencing in transformed tobacco leaves (Guan *et al*., 2017) in this experiment. Middle leaves of three‐week‐old juvenile *N. bethamiana* plants were used for Agroinfiltration. The detailed procedures are described in previous report (Sparkes *et al*., 2006). The infiltrated *N. bethamiana* leaves were imaged using a Carl Zeiss (LSM510, Germany) confocal microscope 3 days after agroinfiltration.

Co‐IP analysis was performed based on a previous report (Lin and Lai, 2017). Briefly, proteins from 250 mg of tobacco leaves, which continued growth 3 days after agroinfiltration, were extracted in 500 μL of IP buffer, then centrifuged and mixed with 20 μL of anti‐GFP magnetic beads (Sigma). After a 1‐h incubation, the beads were washed five times with wash buffer. Beads were then boiled in 25 μL of 2 × SDS sample buffer and subjected to immunoblotting analysis. The original blot scans are shown in Figure S15.

## Author contributions

C.L. performed most experiments. M.S and A.W. performed vector constructions of *qLTG3‐1*. B.M. and W.W. performed investigation of agronomic traits in the field. C.L. and C.C. designed experiments and wrote the manuscript. M.S. edited the manuscript.

## Conflict of interest

The authors declare no competing financial and non‐financial interests.

## Supporting information


**Figure S1** *bZIP71*,* bZIP73* ^*Jap*^, *qLTG3‐1* ^*Nip*^ expression patterns in different organs of wild type ZH11.
**Figure S2** *bZIP71* expression patterns in flag leaves and panicles of wild‐type ZH11 under cold treatment during the reproductive stage.
**Figure S3** Temperature profile at the experimental station of IGDB at Changping, Beijing, during the periods of August, September, and October in 2012.
**Figure S4** Relative expression of *bZIP71* and *bZIP73* in transgenic lines.
**Figure S5** ABA and soluble sugar contents in transgenic lines and wild‐type plants under normal warm conditions.
**Figure S6** Relative expression levels of ABA biosynthetic genes, invertase gene, monosaccharide transporter genes in (A) *bZIP71* overexpression (71OE) lines, (B) *bZIP71* RNAi (71Ri) lines, (C) *bZIP73* ^*Jap*^ overexpression (73^Jap^OE) lines, (D) *bZIP73* ^*Ind*^ overexpression (73^Ind^OE) lines, (E) *bZIP73* ^*Jap*^ RNAi (73^Jap^Ri) lines, and (F) *bZIP73* ^*Jap*^ and *bZIP71* co‐overexpression (71‐73^Jap^OE) lines as detected by qPCR.
**Figure S7** Distribution of G‐box elements in the promoters of *OsNCED3*,* OsNCED5*,* qLTG3‐1*,* OsMST7*,* OsMST8*, and *OsINV4* genes.
**Figure S8** Sequence alignment of three alleles of *qLTG3‐1*.
**Figure S9** *qLTG3‐1* ^*Nip*^ expression patterns in flag leaves and panicles of wild‐type ZH11 under cold stress conditions during the reproductive stage.
**Figure S10** Relative expression levels of *qLTG3‐1* in flowers of Kasalath and *qLTG3‐1* ^*Nip/Ka/Hj*^ transgenic lines measured by real‐time PCR under normal warm conditions.
**Figure S11** Soluble sugar contents in *qLTG3‐1* ^*Nip*^ overexpression lines and wild‐type plants under natural cold conditions.
**Figure S12** Subcellular localization of qLTG3‐1^Nip^ and qLTG3‐1^Ka^ protein in rice protoplasts.
**Figure S13** bZIP73^Jap^ could not bind downstream genes both at seedling and reproductive stages.
**Figure S14** *bZIP71* and *bZIP73* ^*Jap*^ expression patterns in different organs of ZH11 (*japonica* rice cultivar).
**Figure S15** Original blotting images of co‐IP assay (Figure 6D).
**Table S1** Agronomic traits of *bZIP71*,* bZIP73* transgenic lines and wild‐type ZH11 grown in the field under natural cold stress conditions for plants heading on September 13, 2012, Beijing.
**Table S2** Agronomic traits of *bZIP71* and *bZIP73* ^*Jap*^ co‐overexpression transgenic lines and wild‐type ZH11 grown in the field under natural cold stress conditions for plants heading on September 20, 2012, Beijing.
**Table S3** Agronomic traits of *bZIP71*,* bZIP73* transgenic lines and wild‐type ZH11 grown in the field under normal warm condition for plants heading on August 17, 2012, Beijing.
**Table S4** Agronomic traits of *qLTG3‐1* ^*Nip/Ka/Hj*^ overexpression transgenic lines and wild‐type Kasalath grown in the field under natural cold stress conditions for plants heading on September 3, 2012, Beijing.
**Table S5** Agronomic traits of *qLTG3‐1* ^*Nip/Ka/Hj*^ overexpression transgenic lines and wild‐type Kasalath grown in the field under natural cold stress conditions, for plants heading on September 12, 2012, Beijing.
**Table S6** Agronomic traits of *qLTG3‐1* ^*Nip/Ka/Hj*^ overexpression transgenic lines and wild‐type Kasalath grown in the field under normal warm conditions for plants heading on August 22, 2012, Beijing.
**Table S7.** Primers used in real‐time PCR analyses and vector constructions.

**Table S8** One way anova test for *P*‐value.
