# Supplementary material for: The bZIP73 transcription factor controls rice cold tolerance at the reproductive stage
Source: Plant Biotechnol J. 2019 Mar 12;17(9):1834–49. doi: 10.1111/pbi.13104 (PMC6686130; doi:10.1111/pbi.13104)
Supplement: Supplementary file 1 — Figure S1 bZIP71, bZIP73 Jap , qLTG3‐1 Nip expression patterns in different organs of wild type ZH11. Figure S2 bZIP71 expression patterns in flag leaves and panicles of wild‐type ZH11 under cold treatment during the reproductive stage. Figure S3 Temperature profile at the experimental station of IGDB at Changping, Beijing, during the periods of August, September, and October in 2012. Figure S4 Relative expression of bZIP71 and bZIP73 in transgenic lines. Figure S5 ABA and soluble sugar contents in transgenic lines and wild‐type plants under normal warm conditions. Figure S6 Relative expression levels of ABA biosynthetic genes, invertase gene, monosaccharide transporter genes in (A) bZIP71 overexpression (71OE) lines, (B) bZIP71 RNAi (71Ri) lines, (C) bZIP73 Jap overexpression (73JapOE) lines, (D) bZIP73 Ind overexpression (73IndOE) lines, (E) bZIP73 Jap RNAi (73JapRi) lines, and (F) bZIP73 Jap and bZIP71 co‐overexpression (71‐73JapOE) lines as detected by qPCR. Figure S7 Distribution of G‐box elements in the promoters of OsNCED3, OsNCED5, qLTG3‐1, OsMST7, OsMST8, and OsINV4 genes. Figure S8 Sequence alignment of three alleles of qLTG3‐1. Figure S9 qLTG3‐1 Nip expression patterns in flag leaves and panicles of wild‐type ZH11 under cold stress conditions during the reproductive stage. Figure S10 Relative expression levels of qLTG3‐1 in flowers of Kasalath and qLTG3‐1 Nip/Ka/Hj transgenic lines measured by real‐time PCR under normal warm conditions. Figure S11 Soluble sugar contents in qLTG3‐1 Nip overexpression lines and wild‐type plants under natural cold conditions. Figure S12 Subcellular localization of qLTG3‐1Nip and qLTG3‐1Ka protein in rice protoplasts. Figure S13 bZIP73Jap could not bind downstream genes both at seedling and reproductive stages. Figure S14 bZIP71 and bZIP73 Jap expression patterns in different organs of ZH11 (japonica rice cultivar). Figure S15 Original blotting images of co‐IP assay (Figure 6D). Table S1 Agronomic traits of bZIP71, bZIP73 tra [file PBI-17-1834-s001.docx]

**The bZIP73 transcription factor controls rice cold tolerance at the reproductive stage**

**Liu *et al*.**

[**Supplementary Figures**](#Supplementary Figure 1. Geographic distribution of the USDA mini-core population and the wild rice population. Each red dot represents the location where a sample was collected. Global Positioning System (GPS) information was obtained from as reported pre)

[**Figure S1**](#Supplementary Figure 1. Geographic distribution of the USDA mini-core population and the wild rice population. Each red dot represents the location where a sample was collected. Global Positioning System (GPS) information was obtained from as reported pre)

[**Figure S2**](#Supplementary Figure 2. Sequence comparison of rice and Arabidopsis Group S3 bZIP proteins. NLS, nuclear localization signal. The two definitive domains, the basic region, and the leucine zipper, are indicated by lines. The conserved eight-leucine residue)

[**Figure S3**](#Supplementary Figure 3. Cellular localization and transactivation assays of bZIP73. (a) Cellular localization of the bZIP73Jap (a) and bZIP73Ind (b) protein in rice protoplasts. The fusion protein OsMADS3::mCherry was used as nucleus indicator. BF, bright)

[**Figure S4**](#Supplementary Figure 4. Relative expressions of bZIP73Jap under treatments of cold (4℃), ABA, and H2O2 in shoots and roots of wild-type Zhong Hua 11 rice seedlings. Error bar, standard deviation from three independent experiments. Plants grown in the norm)

[**Figure S5**](#Supplementary Figure 5. Assessing cold stress (4℃) tolerance of bZIP73Jap/Ind and bZIP71 transgenic rice lines. Upper panel, seedlings before cold treatment; lower panel, seedlings immediately after treated in 4℃ for three days. ZH11, wild-type Zhong Hua)

[**Figure S6**](#Supplementary Figure 6. Gene ontology (GO) enrichment of genes precipitated with bZIP73Jap::Flag identified by ChIP-Seq. Number on each bar represent gene numbers in the respective GO term. The enrichment analysis and visualization were performed using th)

[**Figure S7**](#Supplementary Figure 7. Binding interaction of bZIP73Jap to the promoters of ABA biosynthetic genes and POX precursors. (a) In vitro EMSA using G-box sequences from promoters of OsNCED3 and OsNCED5 as probes. Left two panels, wild-type G-box sequences. Ri)

[**Figure S8**](#Supplementary Figure 8. Relative promoter activity of ABA biosynthetic genes and peroxidase (POX) precursor genes under normal and cold conditions in rice protoplasts. The relative fLUC/rLUC ratio indicated by x-axes represents the relative promoter activ)

[**Figure S9**](#Supplementary Figure 9. Relative expression levels of ABA biosynthetic and peroxidase precursor genes in (a) bZIP71 overexpression (71OE) lines, (b) bZIP71 RNAi (71Ri) lines, (c) bZIP73Jap overexpression (73JapOE) lines, (d) bZIP73Ind overexpression (73In)

[**Figure S10**](#Supplementary Figure 10. bZIP73Jap OE (73JapOE) and bZIP71 RNAi (71Ri) lines were hypersensitive to ABA treatment; co-expression lines of bZIP71-bZIP73Jap (71-73JapOE) lines were less sensitive to ABA treatment. Wild-type Zhonghua 11 (ZH11), bZIP73Ind (73)

[**Figure S11**](#Supplementary Figure 10. bZIP73Jap OE (73JapOE) and bZIP71 RNAi (71Ri) lines were hypersensitive to ABA treatment; co-expression lines of bZIP71-bZIP73Jap (71-73JapOE) lines were less sensitive to ABA treatment. Wild-type Zhonghua 11 (ZH11), bZIP73Ind (73)

**Figure S12**

**Figure S13**

**Figure S14**

**Figure S15**

**Supplementary Tables**

[**Table S1**](#Supplementary Table 1. bZIP genes correlated with the low-temperature seedling survivability (LTSS) phenotype)

[**Table S2**](#Supplementary Table 2. The list of bZIP genes with excessive LD and their transcription response to cold stress)

[**Table S3**](#Supplementary Table 3. Allele frequency of the functional polymorphism (FNP) of the bZIP73 gene in different rice ecotypes.)

[**Table S4**](#Supplementary Table 4. Nucleotide diversity of OsbZIP73 and its flanking regions in different rice subpopulations.)

[**Table S5**](#Supplementary Table 1. bZIP genes correlated with the low-temperature seedling survivability (LTSS) phenotype)

[**Table S6**](#Supplementary Table 2. The list of bZIP genes with excessive LD and their transcription response to cold stress)

[**Table S7**](#Supplementary Table 3. Allele frequency of the functional polymorphism (FNP) of the bZIP73 gene in different rice ecotypes.)

**
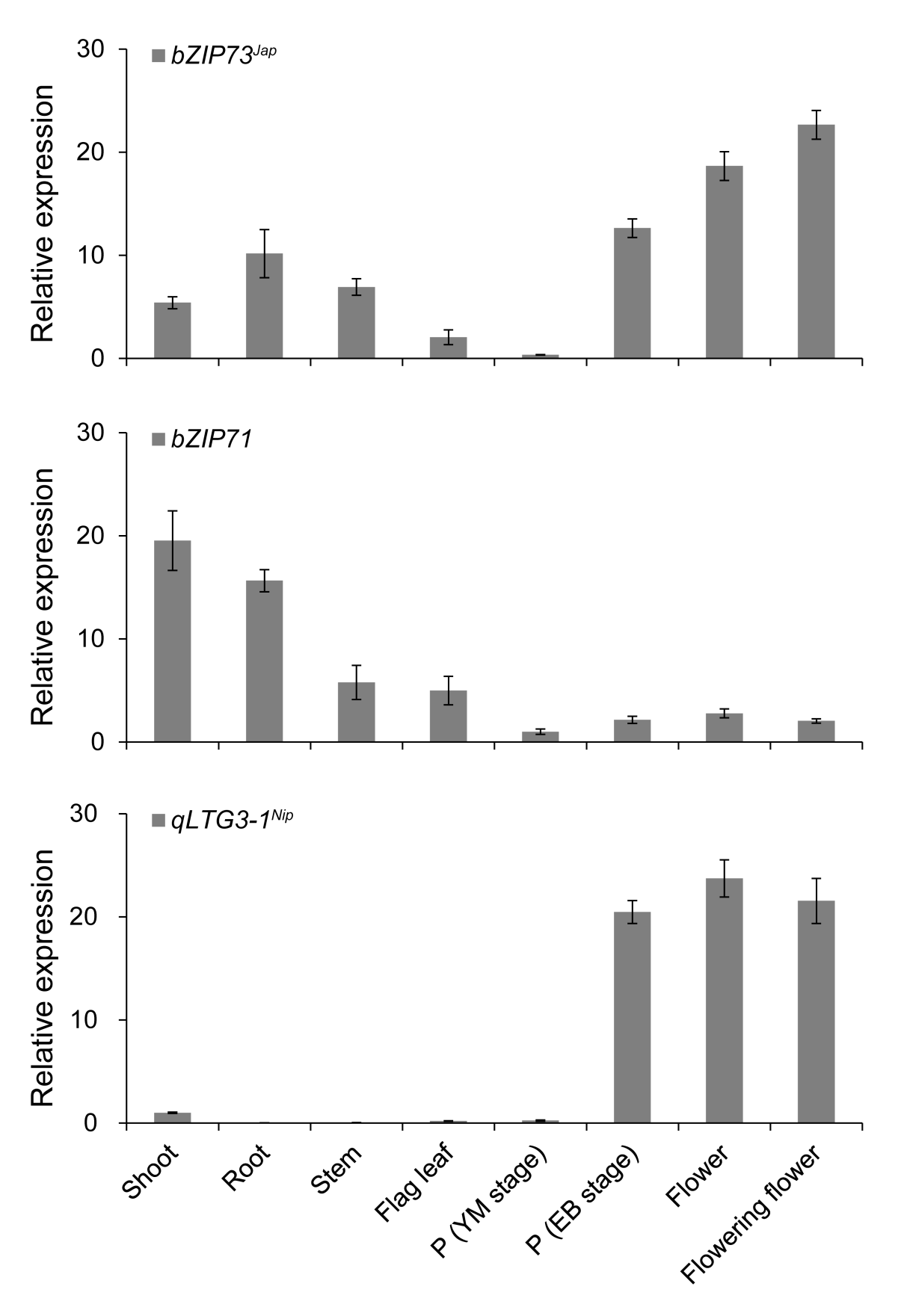
**

# **Figure S1** *bZIP71*, *bZIP73^Jap^*, *qLTG3-1^Nip^* expression patterns in different organs of wild type ZH11. Shoot (two-week-old), root (two-week-old), stem (YM stage), Flag leaf (YM stage), panicles at YM and EB stages, flower (one day before flowering).

**
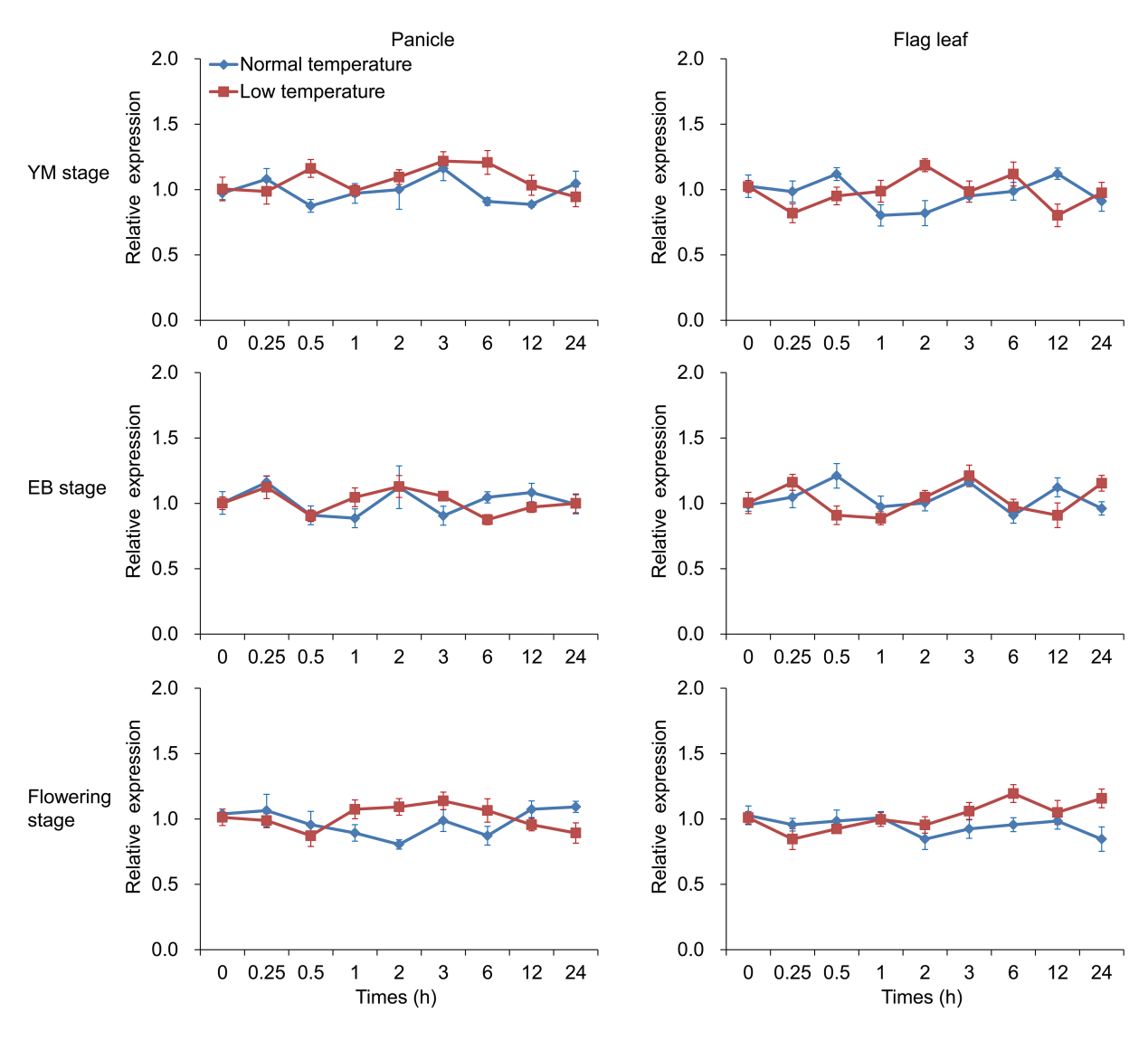
**

# **Figure S****2** *bZIP71* expression patterns in flag leaves and panicles of wild-type ZH11 under cold treatment during the reproductive stage. YM stage, young microspore stage. EB stage, early binucleate stage. Plants grown in the normal condition were used as untreated control. Error bar, standard deviation from three independent experiments.

**
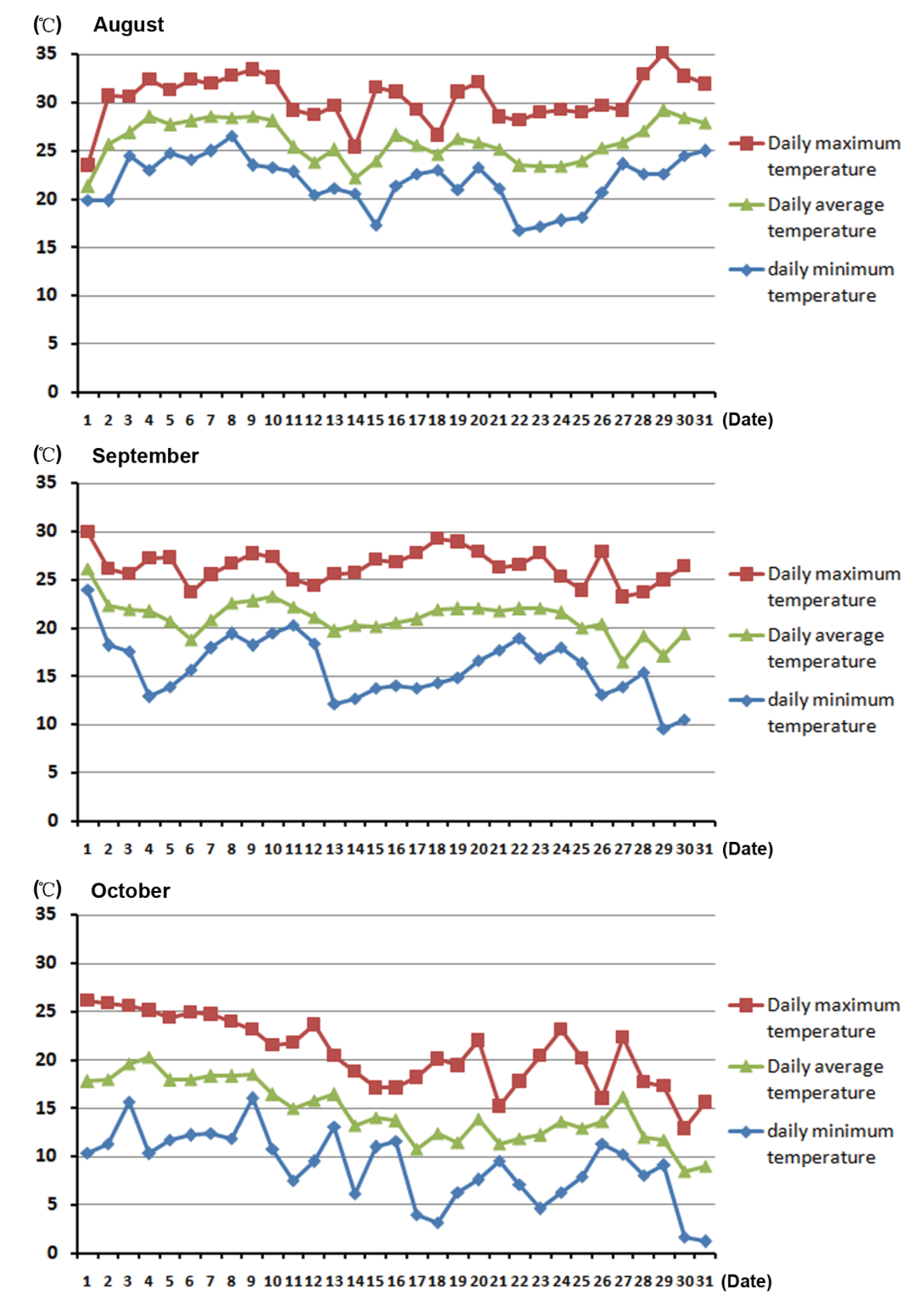
**

# **Figure S3** Temperature profile at the experimental station of IGDB at Changping, Beijing, during the periods of August, September, and October in 2012.

**
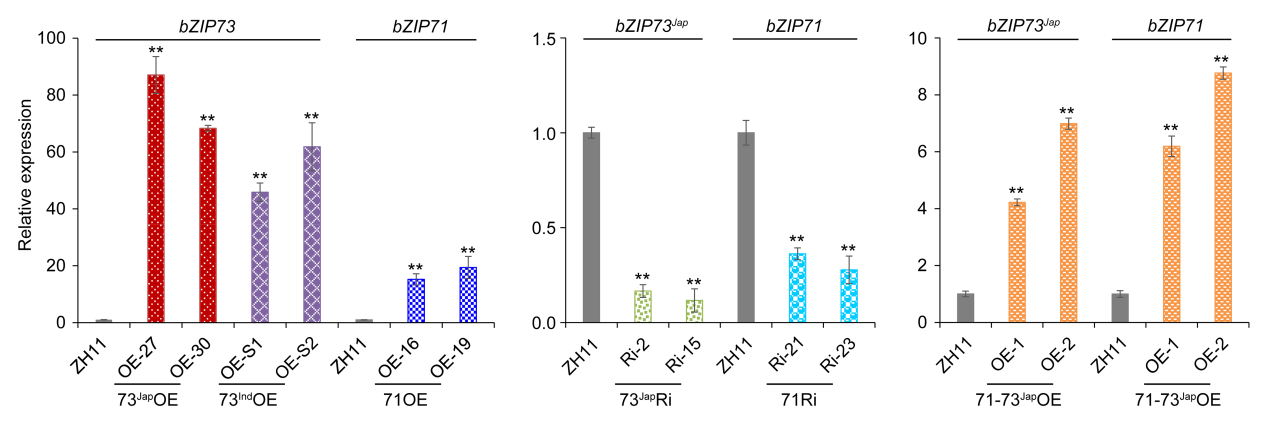
**

# **Figure S4** Relative expression of *bZIP71* and *bZIP73* in transgenic lines. OE-16 and OE-19 are two independent homozygous lines from the *bZIP71* overexpression (71OE) lines; OE-27 and OE-30 are two independent homozygous lines from *bZIP73^Jap^* overexpression (73^Jap^OE) lines; OE-S1 and OE-S2 are two independent homozygous lines from *bZIP73^Ind^* overexpression (73^Ind^OE) lines; Ri-2 and Ri-15 are two independent homozygous lines from *bZIP73^Jap^* RNAi (73^Jap^Ri) lines; Ri-21 and Ri-23 are two independent homozygous lines from *bZIP71* RNAi (71Ri) lines; OE-1 and OE-2 were two independent homozygous lines from co-overexpression of *bZIP71* and *bZIP73^Jap^* (71-73^Jap^OE) lines; ZH11, wild-type Zhonghua11. Each experiment was done using three biological replicates. Error bar, standard deviation. ***p* < 0.01, two-tailed *t*-test in comparison to ZH11.


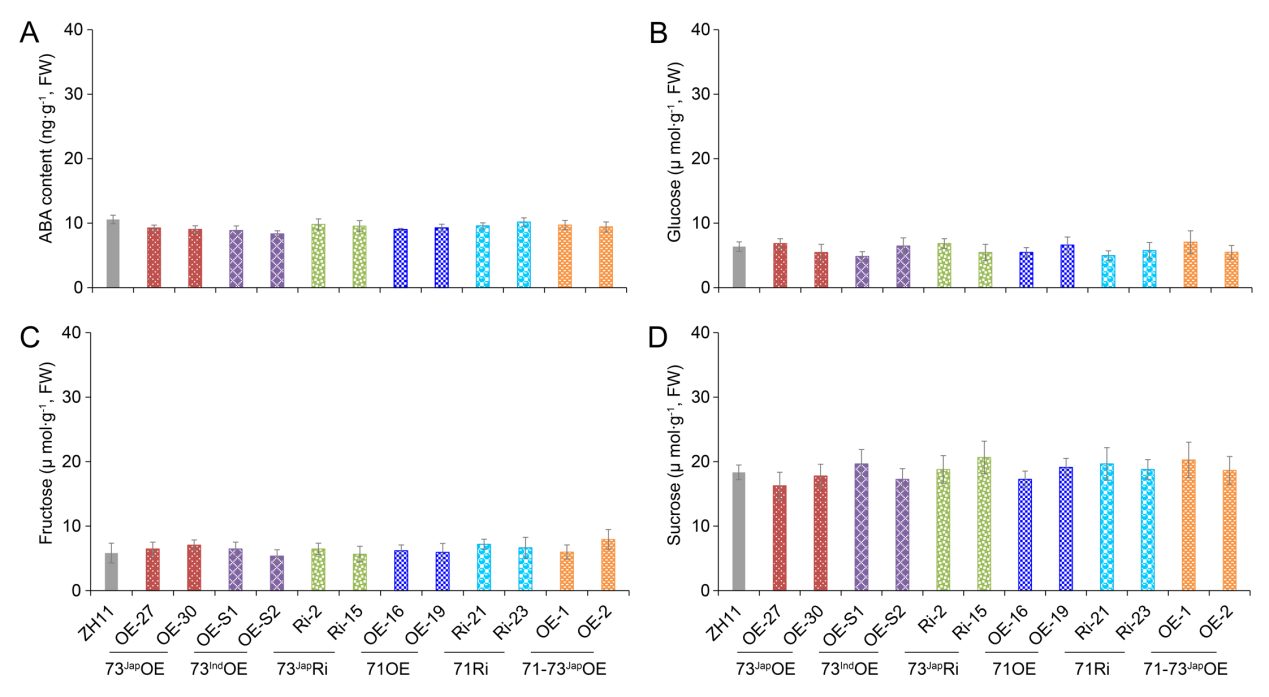


# **Figure S5** ABA and soluble sugar contents in transgenic lines and wild-type plants under normal warm conditions. ABA (**A**) (three biological replicates), glucose (**B**), fructose (**C**), and sucrose (**D**) contents of transgenic lines and wild-type ZH11 under cold stress conditions. FW, fresh weight. All experiments were conducted using flowers one day before heading. Values are the mean of ten independent biological replicates. Error bars indicate SD. One way anova test.


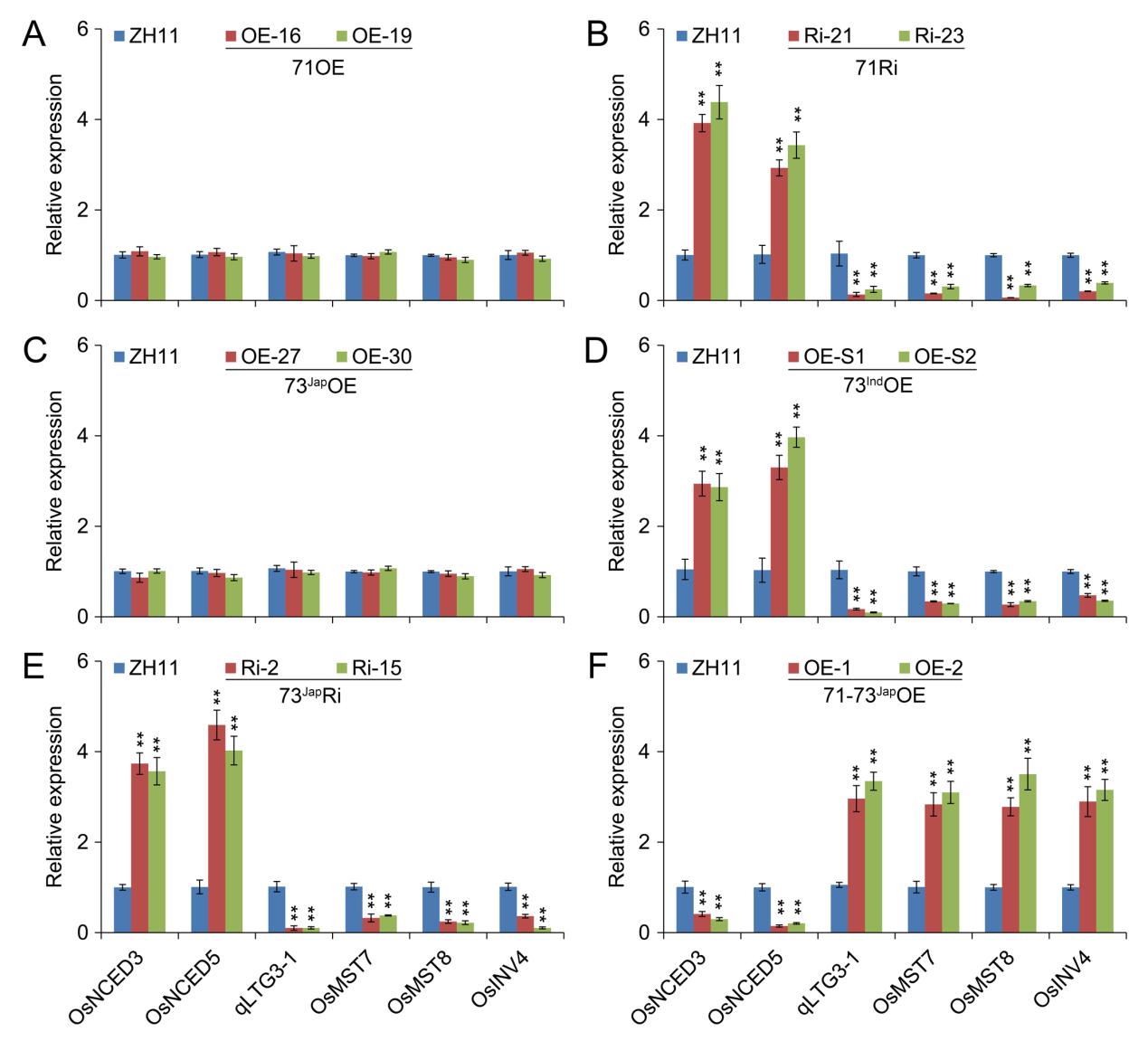


# **Figure S6** Relative expression levels of ABA biosynthetic genes, invertase gene, monosaccharide transporter genes in (**A**) *bZIP71* overexpression (71OE) lines, (**B**) *bZIP71* RNAi (71Ri) lines, (**C**) *bZIP73^Jap^* overexpression (73^Jap^OE) lines, (**D**) *bZIP73^Ind^* overexpression (73^Ind^OE) lines, (**E**) *bZIP73^Jap^* RNAi (73^Jap^Ri) lines, and (**F**) *bZIP73^Jap^* and *bZIP71* co-overexpression (71-73^Jap^OE) lines as detected by qPCR. ***p* < 0.01, two-tailed *t*-test. Error bar, standard deviation of three biological replications (10 plants/replication).

***OsNCED3 (LOC_Os03g44380)***

GCCGGGCACCGCAGCGAATTACGACTTGGGAGGGCGTGCTGGCCTTGTCTGACTGTCCTCGTACGCGAACAAGCCTCAGCGTTCGTCCCTTGTCGACGATGGTCGGTGGTGGGTCGGCCGATGGCGCTTTGTCGATCACCTCGGCGCTCACTCAGCACGCGCGTTTCGCTTCAATTCTCACACACGAGACTGTCCGTACGCCGGGGCTCTCGTGTTGTGCCAGCACCGAAGGTTTAGTACGTCCTGTATCTCGTCCCTGTCAATGTCTGCGGCGCCGGCGGCTGCGGTGTTTGTTACTGTGTCCCATGCAAACAATCTTGGTGATGAGAAACCTTGGTAGTATCGTTGCCAAGGAGGGGGGGTTTATCTCCCCGGGGGAACAGAACACTCCAGGGCGCAAGTACCGACCGCCGCCAGCGCCAGGAACAACGGGAGACTCGATCGTGAGTCGCCACTCGATACACTAGTAAAATATAAAATCCATAATCCACAATCCAAGCTGTGC**AAGCTGAACAGCAGCCAAG**GAGGAGGGAGGGGGGAGATCGAGCGGGGGGCCCCCTCGCGAGTCGCGGGTGTTTTACTCGCGTCGCGCGGTCGCCGTCCCCGCGTCGCCCTGCCTCCCCCCGGAGCGCAC**CACGTG**GGCACCCAACCCCCTCTCCCAGCCCAAAGCAAATTCTGTCAACTTCAAGCTTGGGAGGAGGAGGAGAGTTGGACCCCTCCC**CGTTCAAACCGCCCACG**CCCCCGTCCCCACCATCACTCCACCCCCCCCCCCCCCCCCCTCCAACTTGCCAATGCGCCTCTCCCTACCTCCTCCTCCGTCTCCCCTGCTCTCCTCTGCTCCGTTAACTCTATATATACGCTCACTGGCTACCTCCCCTCCCAAACCATCCAAACCGACGAGCTCTCTTCCTCTCTCTCCAGCTCGGTCTCACACACACTCTCTCTCTCTCGGCAGAAACACACCACGATCGCGGCCAAAACCCACGCGATTGTAGCTCAGATTGTTGTGCGATCGACGCGAT**ATG**GC

***OsNCED5 (LOC_Os12g42280)***

CAATAAATGTGGTGAAGGAAAACTCCACTGCTAAATCTAATTTCACGATCGAATTCTGAAATTCAAAATGATATATAGAAATGTTAGAAACAGCGCCAAGCGTCTTCTAAATCTCCCAGGCGCTTAAAACTTAGCTTGATAAAATAAATCCTTAAAAGTTTCATGCTTTACGCTCAATACACCCACCCAACTAATTTTCAATTATAGGAGCCTGCCCATTGCAAAGCCCAGGTTGTATATTACTAATAACAGAAAAATATAAGAGAGAACTGATAAATAAGAGTAAACAAACTAAAGAGAGGAAAAGAGAGAAAAAAATAAAGAGAATAAAGGAAAATTGAGGCAAACAATAAAAATGACGAGGGCATAAGAAAAGAAGTAAAAGTAGAAATAAAATAAAATGAGAGAGAACAGAAAAGAAATAAAATAAAATAGAGAGATTACATATTAATAATAAAAAAAAAAGAGGGACTTGAATGAAGAAAAGGAGAGAGCATAGACATAAAAAGAAAGAGAGGGGGAAGGAGAGAAAAGAAATAATGAGAATAATAGAAAATTGAAGAAAAAATAAAAGTAAAGAAACACAAGCAAAGAAGTAAAAACACAAATAAAAAGAGAGAGAACAGAAAAGAAAGAGAGGGAATTGAAAAAGAAGAAGAAAAATAAAAATTAGTAAAAAGAGGAATAGAGGCGTC**AGTGTCCAAGTCCACTGAG**CTCTGCTCTTCTCTCCGGCGAATCCAC**CACGTG**GTCTCCTCTCCCAACTTCACCTCCTCCTCTTCCCCC**CTCCTCCCTCTCCTCCT**CCTCCCCTGCATCCACCTCCCCTCCCCCTTCCCGCCGCTGTCTCCTTCCTCCCCCAAACTCTATATATACCCTCCCCCCTCCTCTCCTCCTCTCCTCATCTCATCACCCACCTCCTCTTCTCCCGCCAACTCCAGCTCTCACCTCTCCTTTTCAAGAATCCATCCAAGAATCGATCGAGCACCTTGTTCTGCTCTTGTAGGACAGCC**ATG**

***qLTG3-1* (*LOC_Os03g01320*)**

TAGAATGTGTCACATCCGGTACTAGCTAGGTTGGTTTTTATGGGACGGAGGGAGTATATATGTGACTTATCGATGAACTTTTTCGGTTAATTAGACGATGAGAAATGATTTTATGTGAAGTTAATTAATTAATTAACCTCTAACATTTAACTAATGCAGGTAATATATTATCTTATAGTTAATTAGACGCTAGAAAATGGTTTAAGATTGATCGAAACCAATATTAATTAATGAGGTCTATTTATAAAATTAATTCAGTTGCAATATATACACGCTCATTTAGCTAGTCTCCAAAATATATCGAAATAAATATCGATTGGCCCTTGCCTCCCGCAGGTATATTATTG**CATATTCCCTCCG****TCCCAAA**AAAATAAACCCTGGGTTTT**CACGTC**CAATATTTGACTGTTTGTCTTATGTGAAATTTTTTTTATAATTAGTATTTTTATTGTTGTTAGATGATAGAACATGATTAATAT**TTTATGCGCGACTTGTCTTT**TAATTTTTTTTTCATATTTTTTTTAAATAAGACGGACGGTCAAACGTTGGACACGGAAATCAAGGGTTTGTCTTTTTTTTTTGGGGACGAAGGGTGTATATATATATATATATATATATATGCTCGCTAGCAGAGTACTTGGCCTCCAACTCAATCGTCCTTGCGGCCAGCTAAGCTACCAAAAGCCCACTAGCTAGTAGCACTATACTCCAATGAATTAATGTACTCCAGGCCAGCTAGCTAGCTAGGTCACTAGTAGTAGTTACGCTGTAGTGATGTGAGCTGCACCTAAGATGAAATCCTAGTTAAAATCCGATTAGTTAATTAGTTAGTTAAGTGATGTGGTAGCTGGTTAAGTAATTGAAGGTACTCCGATCCTATTATAAATATGAGTGCACCCCTTGGTCCATAGATGTATCTGAATACACTGGCTAGCAAGCAAAGCTAGGTAGAGGCCAGGCCATAGATCGAGATCGATCTGCAGGTGCAGTGCGGTGGGTGGGC**ATG**GCG

***OsMST7* (*LOC_Os01g38680*)**

CTTTTTTTTTTGTTTTTTTTCGCTTTTCTTATTTAAACGGTATTTTTTTCTCGGACTTTTTTTTCCCTTTTAAGGGATCGGATATTTTTTTCCCCAGGGGAATCGGACTCTTTTTGTTTTGTTTTTTTTTTTCGCTTTTTCTTATTTATTAAACGGACGAAGGAAACGTTTCTTACTTTTTAAGTAGTAGTAGAGATAGATATTATTATTATTATTATTATTATTATTATTATTATTATTATTATTATTATTATTATTATAAGTAGTAGAGAAGATAGAGATAGAGAATCGGATTTTTTGAAAAAAAATTTCGCCCTTTTTTACCGCGTTGACGGAATCAGATTCGTTTTTCTTTTCCCTTATTTTTGCCTTTTATTGGATCGGACATGTTTGTTTTTTTCTTTTTTTCGCCCGGATTTTTTTTGGATCGGACTGATTTGTTTTTTTTTTCTCCCGGTTTTTTCGACGGACGATGGAAGTACCTTTTTATTTTTTTAAGTACTAGAGAAGAGATAGAGAAGAATGTGCTTGTCAATTCTGGTGGGGTGTCGCTACTTCGATTCCTTGGTGGAACGATTACATGAAACTAGCGGTTCATTGATCTTCGTCCATCGTACTGGGGTTAGTGG**GGTGGATTATGTTATCTCTACCATGT**ATATTTTAATTTGAAAAAATAATGGGTAGAAAAAACTATCAATTA**TACGTG**ACGAAACATTGAGATATATGTATTAG**AGAGATTAGTTTGAATAAGCCAGG**ATTAATTAGATGTCCTCGTTAAGGATGAGGAAAAATGTAATAATTTGTACTACGCGGTAACCTATGAGAAGGATAAACTTAATCATAACCCAAATGACCTGAAGATATTTAATGTAAAATTTTATCTCTAATGATATCTTAACCGTTTTATAAGCCCTATATAAGCTGAAGCGAAGCCA**CACGTA**CCGTATCGACACAACTCCTTTCCCATCGATCTGATCTCCCGTGTCCCTTCTCTCGTTTCCCTGGCA**ATG**G

***OsMST8* (*LOC_Os01g58670*)** TAGAGGCACTTCACCGTGGGTTGCCACCCACCACCCTGCTAAAGAAAGAGTGAAGAGAGGAGTGGGGAGACATGGAGAAAGAAGGTGAGGGTGGCAACTTACACATGAATCCCACTTACATTGGAGAGGATTTGAGAGACTTACATATAGGTCCACCTTTTTTATTTTTGTTTGCTAATTATAATATCACATAAATGCTATGTCGAAGGAAGACCAAATCAACGTGTCATATAGGACGACACTGTCATTCAAACCGCTTAGGAAGCACAATGAATCAGTTTAAACAGTTAAGGGAGCATCTGTATCTAGTTCGCAAAACGAACCTGTTTAGGTTGTTGAGGGAGCCTCCCAATCTAGTTTTTTGGTTAAGGGATGAACTATACCGAGGAGGCAAAATAGACGTTTTCTTCTTTCTTTTCTTAACGGCCCAT**CACATGTCT**ATAACCCCACTAGCTAGCTCTTTCTTAATACTGGGCTAAAGCCCACACCACAGGCCTACTGAGTGGGCCTAGTGGCACTGAAGTGTGATACTAGTAAAATATCTACATATCGATGATAATACATA**CTGCAGCAGTAGCACTCTATAA**TGTACACTGATCTTGTGCTATAGCCTATAG**GACGTG**CCATACAGTACGAAAAATATCATTTTATATTTTCCTTTC**TTGAGATGGCACATACGC**ACACTAGTAGTATCTCGATCCTGATCAATGCTCCACATCACCGAGAAGCCAGCCTTGCGTCCATGCACCAACGGATCGCGCTACATTTTCTGCAACTAACAGCGCCTAACAACGGATTAGGGATAATATATAGGATCGGATAAATAATTAGTTGGTCACTAGCTGTTACTGTGACTGTGAAAGTCTATCCAATCCAGTAATCCACTCTATATTAACCGTAACCCCCTTCCATTTCTCCCCTGACTGGTGAGTACTGATCGTAGTCCAGCCTTGTTCTCCTCTCTCTCTCTCTCTCCTCACTCCTCGCACACTTCGGAATTCCCATC**ATG**G

***OsINV4* (*LOC_Os04g33720*)**

CTACGGCGGCCGGCCTCCGGGTGGATGAGCAGCTTGAACCTGCCGTCCTCCTTGCACGGCTGCAGCAACTCCTGCGACGGCAGCCGCATCTCCCGCAGCACGAGCCGGCCGGCCTCCCGGTGCGCCCGGAGGCTCAGCCACGGCCGCCCGCCCTTGCCGATCACCGATATCGGCGGCGGGAATGCCCTCCCGCTCCTCGTCCTCCTCAGCACCATCACGGCGTGCTGGTTGTCGTCGTCGTCGTGGCCTACGCCTTCGCTGCTGCTGCTGAGGAGGACGTGGTGCGGCCTCTTGCACGGCGGGGCCAGAACGTCGACCGCGGCGACGGCGGCGCCGACGTCCTCCATGGCGGGGGAGGCGGCGGTGGCGTCGTCGTCGTCGTCCGGATCGAAGCTCTCGGCCCCAAGTGCCTCCGTGAGGAGGTCGAGGCCGCCGGAGTACGGGAGGAGGTGCGGCGGGGGCCTTGATTGTGTGGCGGGGCGCGGCCCGAGCCGCTGCTCCTCGGGGGCTTTGTAGCAGTGCAGCGGTGGTGGCGGTGGTGGTGGCGC**CATGGACAAACGGACGGA**CGGGCGTCGCTCGCGCGGCGGTT**CACGTG**GCGGCTAGCAGAGCGCCCGCACGCGGGTGGGCGCGAGCTATATATATCTGC**GATGGCGAGAGACGAGAC**GGCGAGATCGCGCGGCGCCGATCGATCGAGAGCGAGTCAGCGGGGGCGGATAGGGGAGAACGAAGCGTGCGCGATCTGATCGACCGATCTCTCTCGCGCGCGCGCGTGCCGCTCTTGCACTAGAGATAAGAC**TACGTG**GCACGCCAAAACTGTCCTGAATTCGTGGCAAACGGAAAACGGAAAGAACGTCGCGCGCGGACGCAGCCGCCGCGCCGAGATGAGAAGAAGCTAGGCAAGCTAGAAGCCCCTATAAAATAGTACCATTCACCTCACTATATCATTGATCATCCGAGTCCTAACTGGGTCTCACTAAGCGAGGTAAGTAGTGTGTTAGTGGAA**ATG**GCGA

***Ubiqutin* (*LOC_Os03g13170*)**

**ATG**CAAATCTTCGTGAAGACCCTGACTGGGAAGACCATCACCCTCGAGGTGGAGAGCAGCGACACCATCGACAATGTCAAGGCTAAGATCCAGGACAAGGAGGGAATCCCGCCGGACCAGCAGCGGCTGATCTTCGCCGGGAAGCAGCTGGAGGACGGACGCACCCTGGCTGACTACAACATCCAGAAGGAGTCCACCCTCCACCTCGTCCTCAG**GCTCCGTGGCGGTATCAT**CGAGCCGTCGCTTCAGGCGCTTGCCCGCAAGTACAACCAGGACAAGATGATCTGCCGCAAATGCTATGCGCGCCTGCACC**CTAGGGCTGTCAACTGCCG**CAAGAAGAAGTGTGGTCACAGCAACCAGCTGAGGCCCAAGAAGAAGATCAAGAACTAG

# **Figure S7** Distribution of G-box elements in the promoters of *OsNCED3*, *OsNCED5*, *qLTG3-1*, *OsMST7*, *OsMST8*, and *OsINV4* genes*.* The 1-kb promoter sequences were retrieved from TIGR database (http://rice.plantbiology.msu.edu). G-box elements are shown in red color. Sequences in blue color and underlined indicate the primers used in ChIP-qPCR assays. Sequences in yellow color indicates the primers used in EMSA assays.


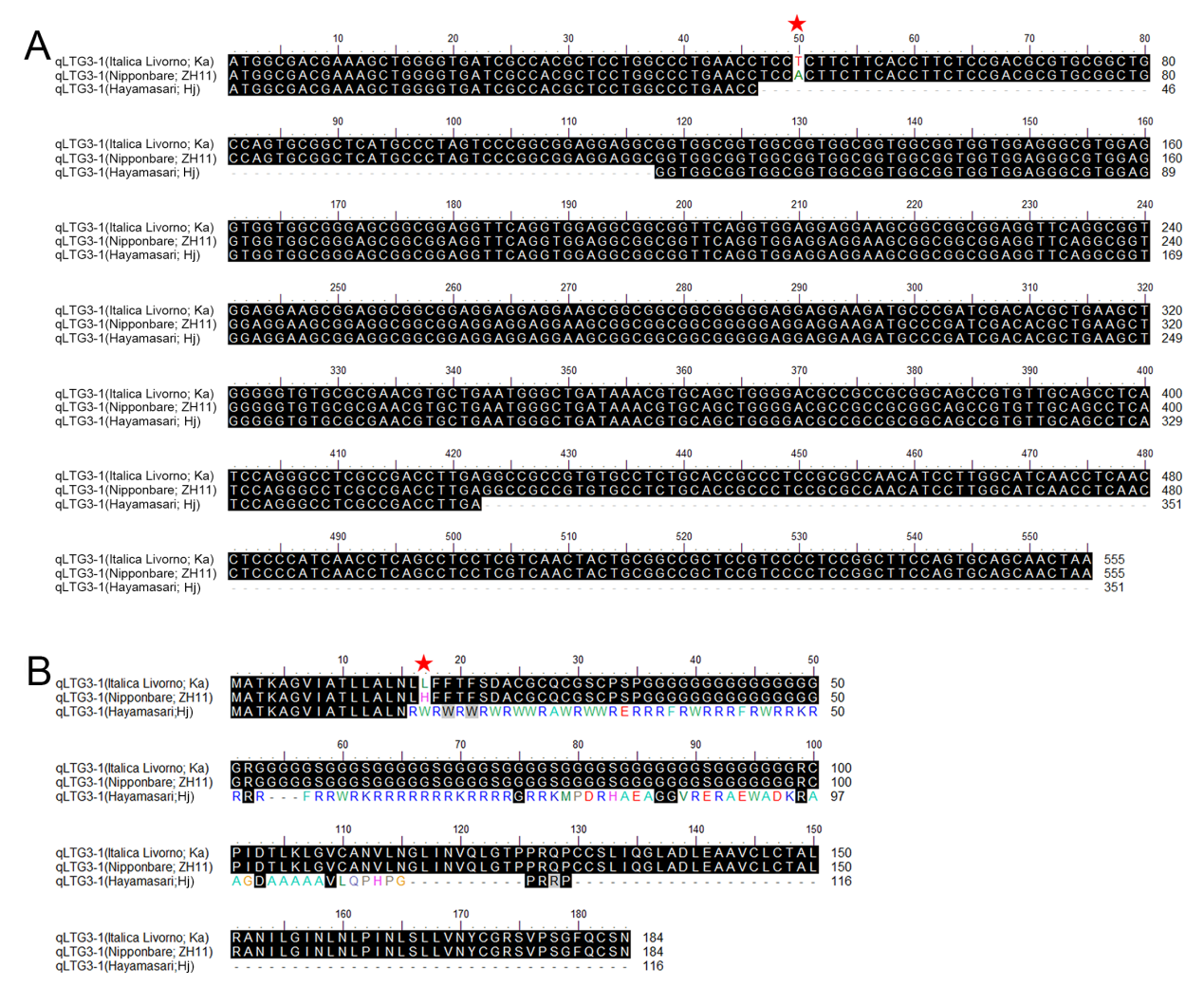


# **Figure S8** Sequence alignment of three alleles of *qLTG3-1*. (**A**) The CDS sequence alignment of three alleles of *qLTG3-1*. (**B**) The protein sequence alignment of three alleles of *qLTG3-1*. Ka: Kasalath; ZH11: Zhonghua11; Hj: Hejiang19.


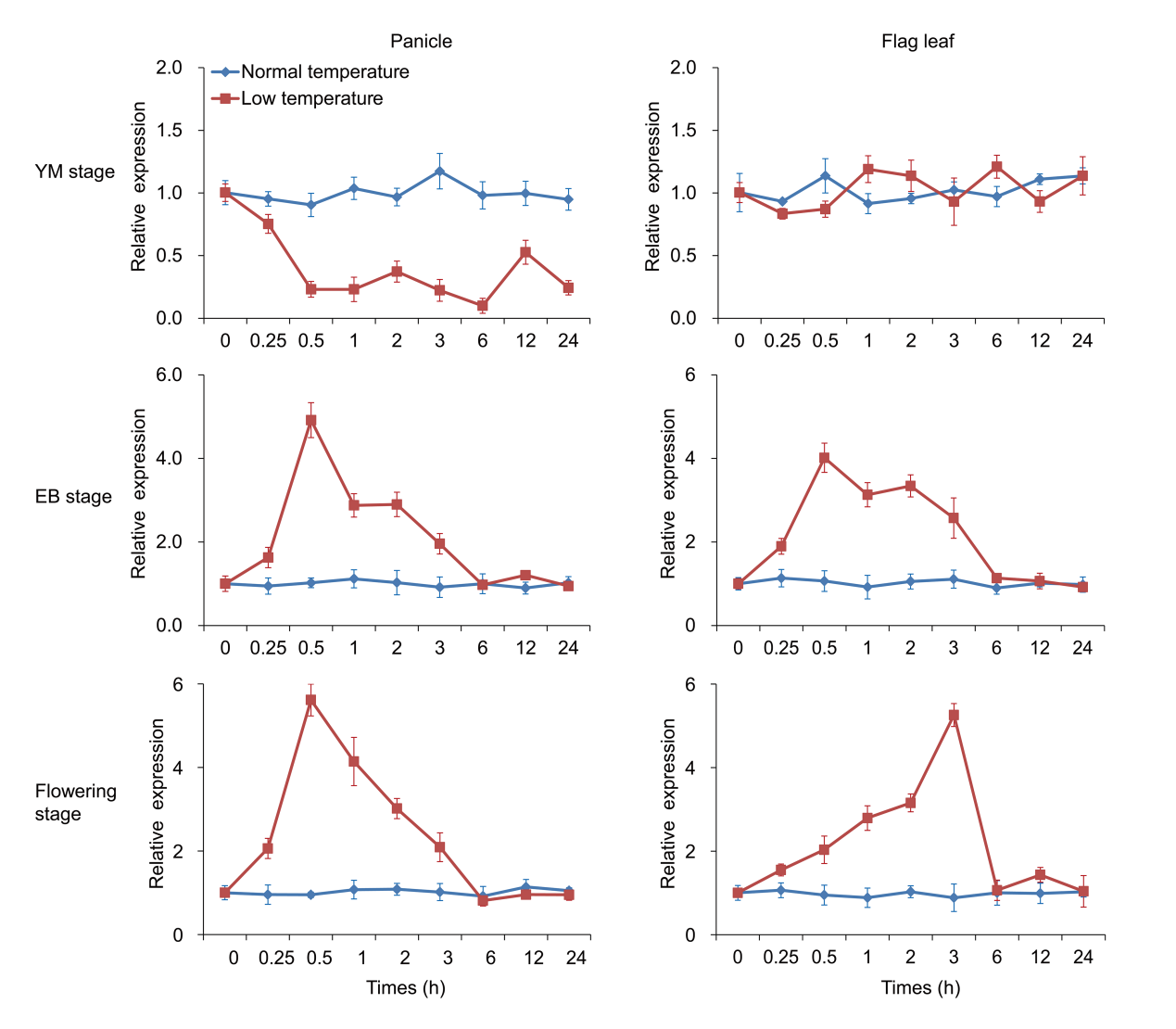


# **Figure S9** *qLTG3-1^Nip^* expression patterns in flag leaves and panicles of wild-type ZH11 under cold stress conditions during the reproductive stage. YM stage, young microspore stage. EB stage, early binucleate stage. Plants grown in normal warm conditions were used as untreated control. Error bar, standard deviation from three biological independent experiments.


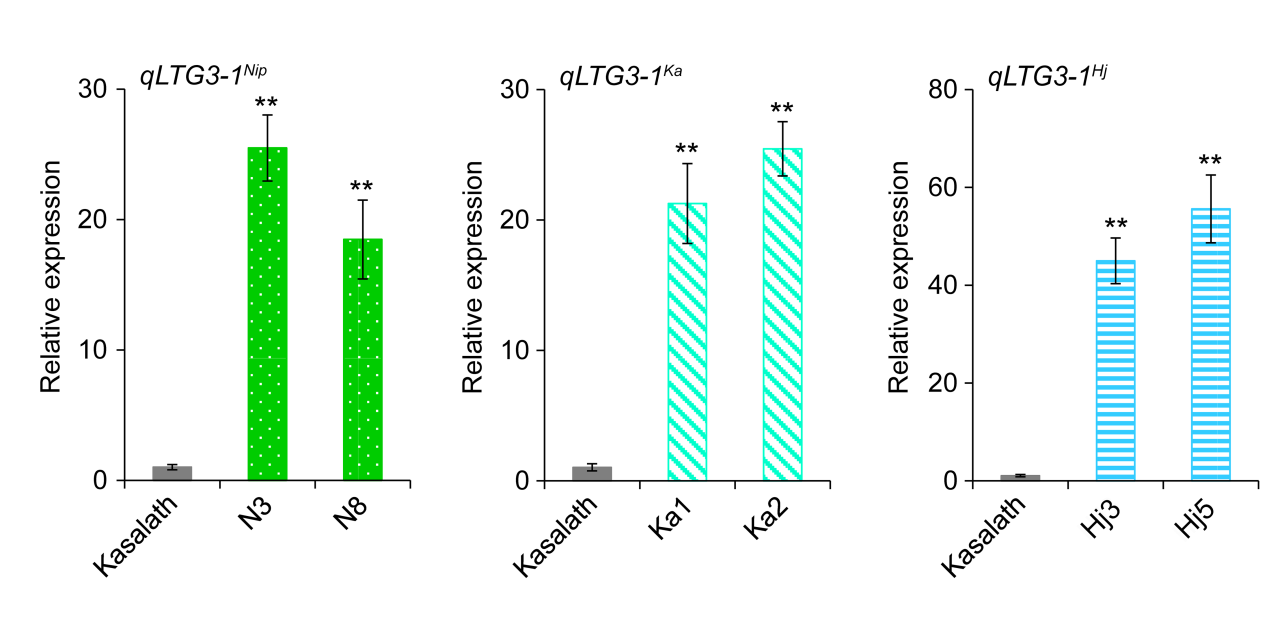


# **Figure S10** Relative expression levels of *qLTG3-1* in flowers of Kasalath and *qLTG3-1^Nip/Ka/Hj^* transgenic lines measured by real-time PCR under normal warm conditions. N3 and N8 are two independent homozygous lines from *qLTG3-1^Nip^* overexpression lines. Ka1 and Ka2 are two independent homozygous lines from *qLTG3-1^Ka^* overexpression lines. Hj3 and Hj5 are two independent homozygous lines from *qLTG3-1^Hj^* overexpression lines. Each assay was done using three biological replicates. Error bar, standard deviation. ***p* < 0.01, two-tailed *t*-test in comparison to Kasalath.


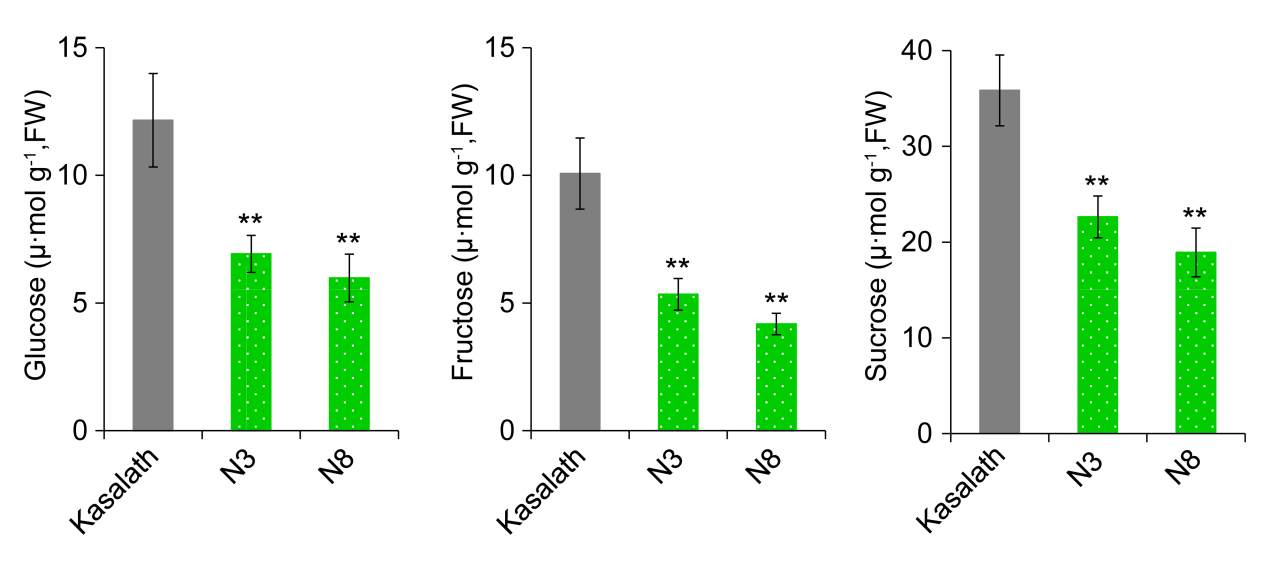


# **Figure S11** Soluble sugar contents in *qLTG3-1^Nip^* overexpression lines and wild-type plants under natural cold conditions. Glucose, fructose, and sucrose contents of transgenic lines and wild-type ZH11 under cold stress conditions. FW, fresh weight. All experiments were conducted using flowers one day before heading. Values are the mean of ten independent biological replicates. Error bars indicate SD. ***p* < 0.01, two-tailed *t*-test in comparison to Kasalath.


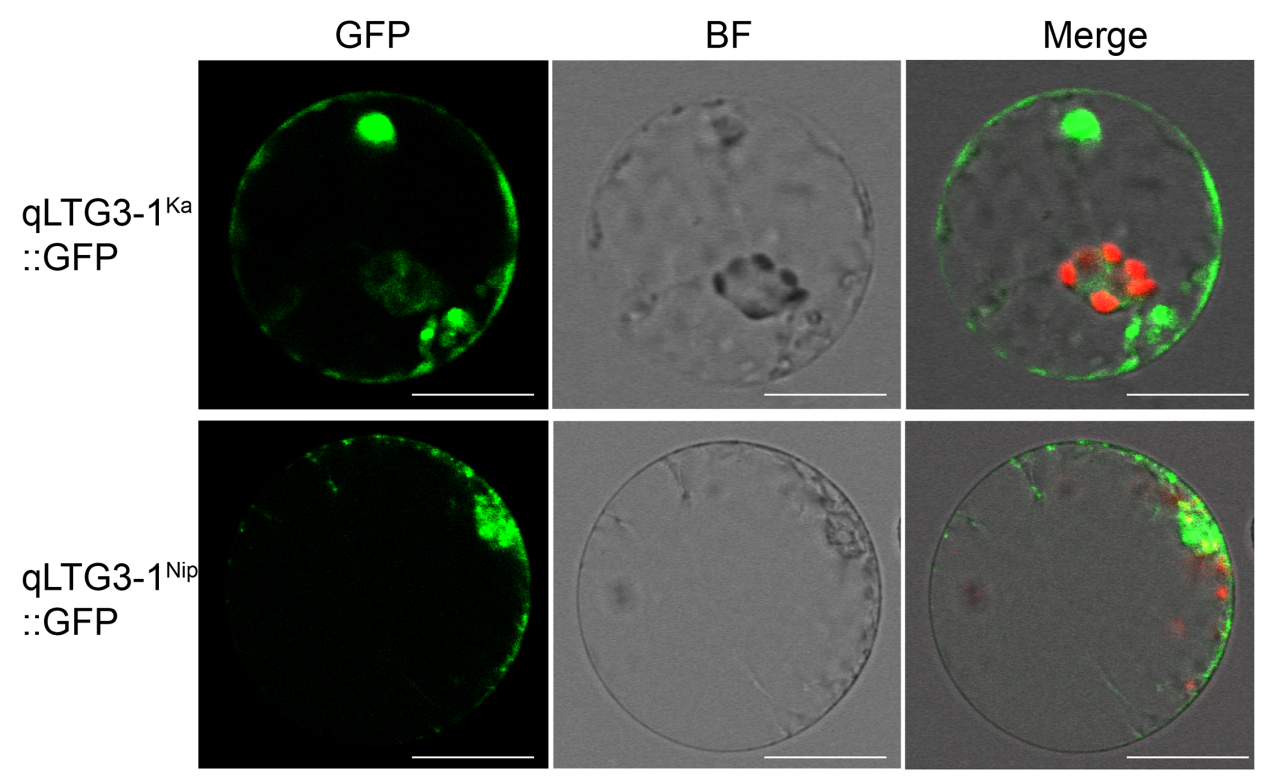


# **Figure S12** Subcellular localization of qLTG3-1^Nip^ and qLTG3-1^Ka^ protein in rice protoplasts. GFP fluorescence (left); BF, bright field (middle); Merge, overlay of the GFP and bright field images (right). Scale bars = 10 μm.


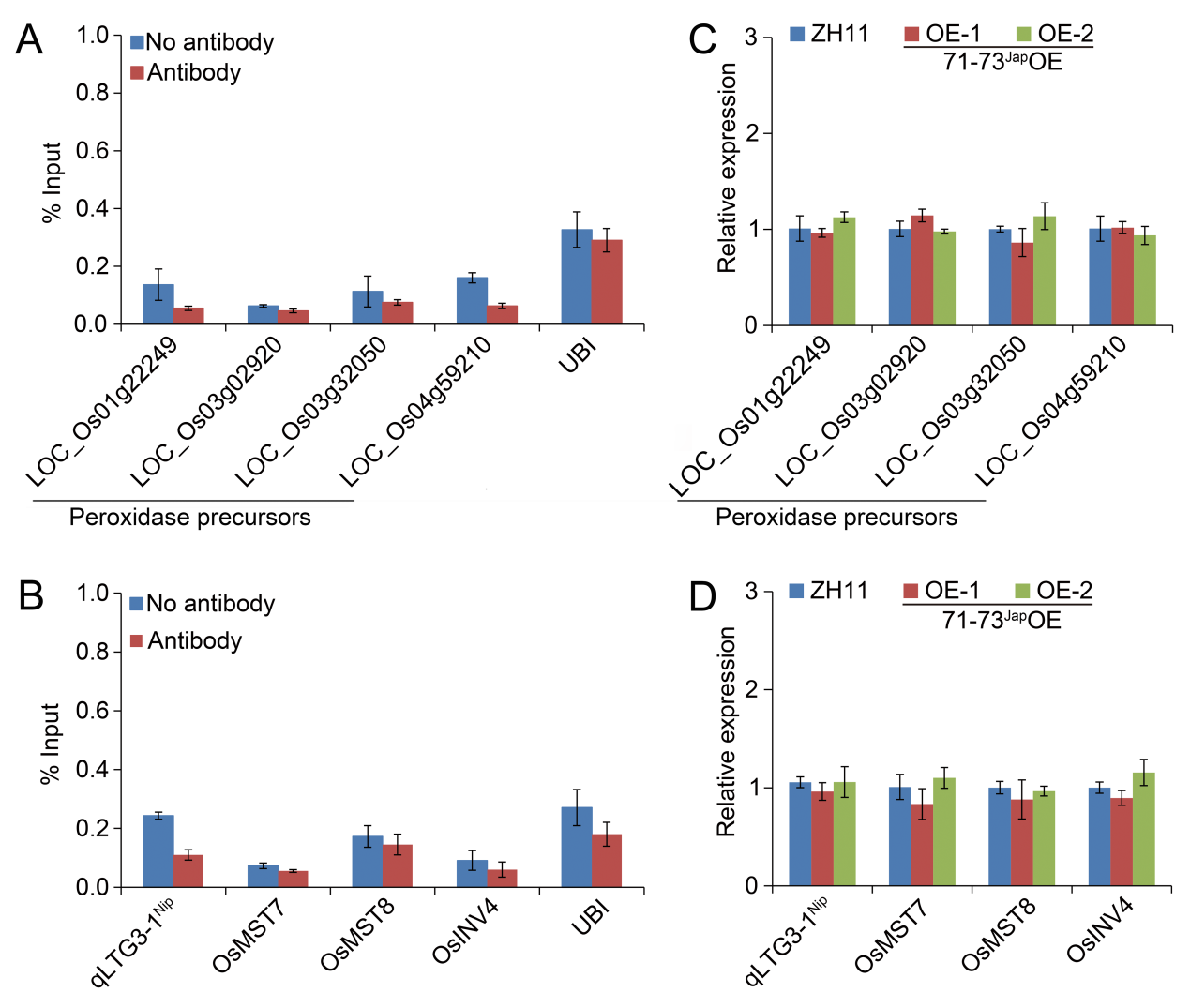


# **Figure S13** bZIP73^Jap^ could not bind downstream genes both at seedling and reproductive stages. (A-B) ChIP-qPCR using bZIP73^Jap^::Flag overexpression lines with three biological replicates. Flowers in reproductive stage (A); Shoots of seedling (B). The anti-Flag antibody was used to precipitate bZIP73^Jap^-DNA interactions. Precipitated DNA was amplified with primers overlapping the G-box motif. ChIP, chromatin immunoprecipitation. Error bar, standard deviation. two-tailed *t*-test. (C-D) Relative expression levels of downstream genes in *bZIP73^Jap^* and *bZIP71* co-overexpression (71-73^Jap^OE) lines as detected by qPCR. Flowers in reproductive stage (C); Shoots of seedling (D). Error bar, standard deviation of three biological replications (10 plants/replication).


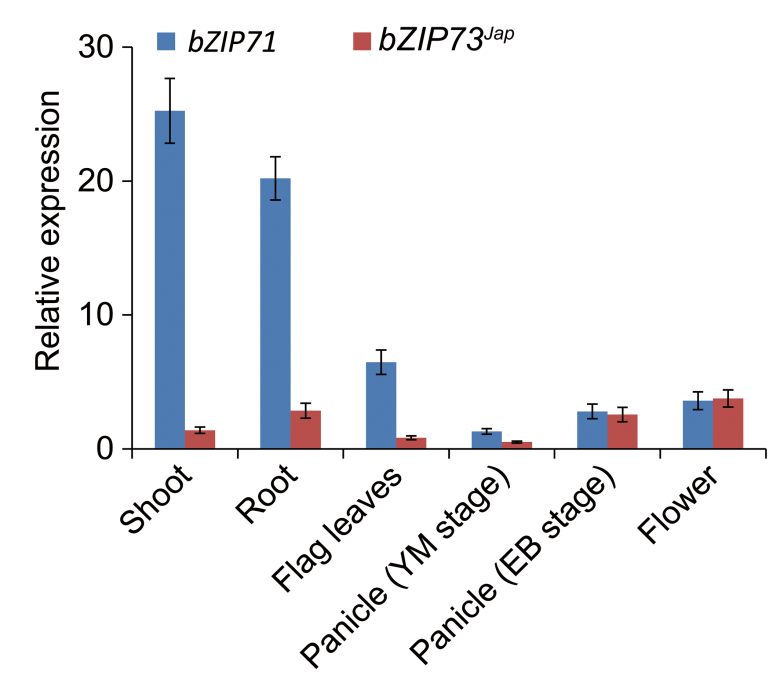


# **Figure S14** *bZIP71* and *bZIP73^Jap^* expression patterns in different organs of ZH11 (*japonica* rice cultivar). Shoot (two-week-old), root (two-week-old), flag leaf (YM stage), panicles at YM and EB stages, flower (one day before flowering).


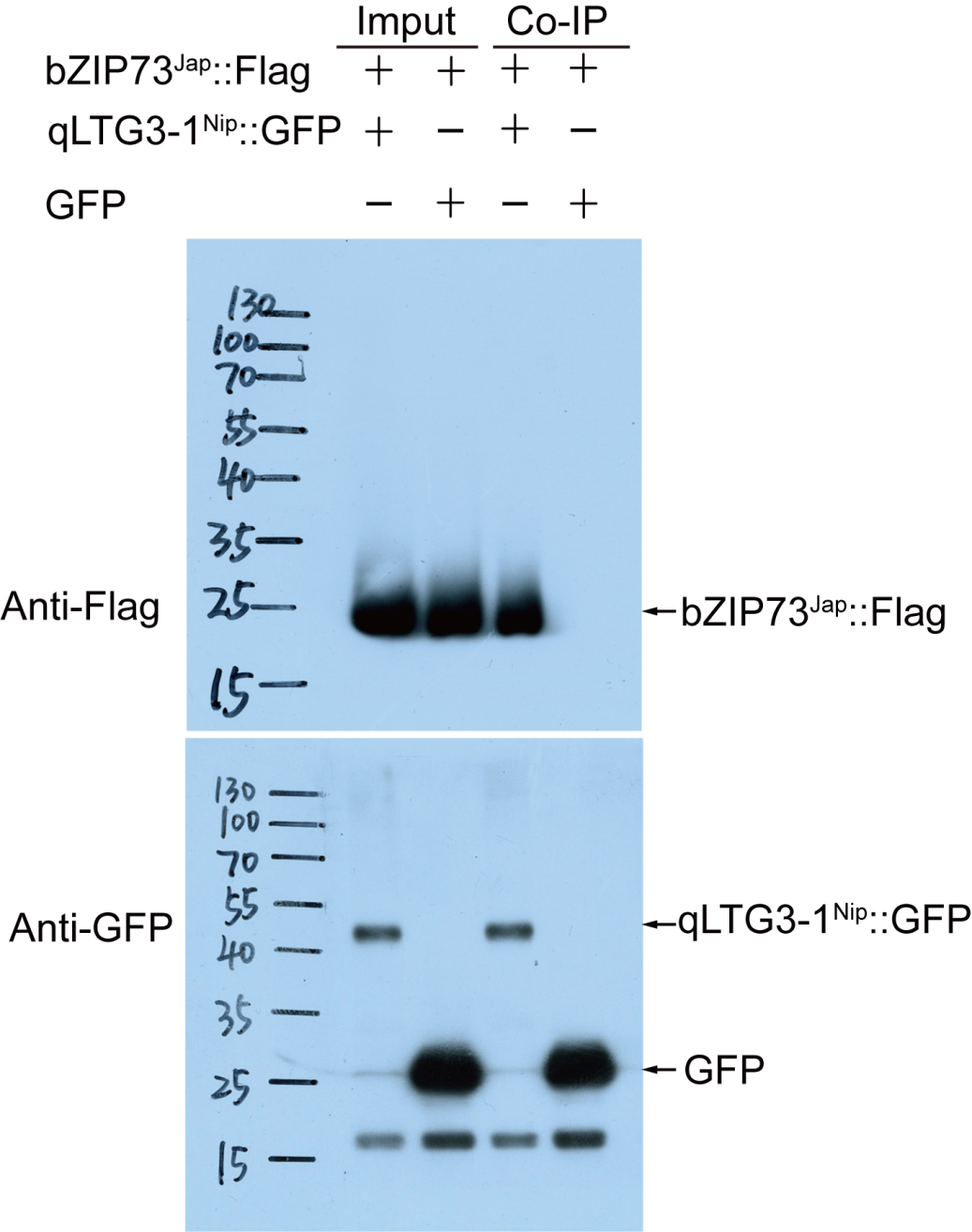


**Figure S15** Original blotting images of co-IP assay (Figure 6D).

# **Table S1** Agronomic traits of *bZIP71*, *bZIP73* transgenic lines and wild-type ZH11 grown in the field under natural cold stress conditions for plants heading on September 13, 2012, Beijing.

| Line | Till numbers | Panicle length (cm) | Seed setting rate (%) | Yield per plant (g) |
| --- | --- | --- | --- | --- |
| ZH11 | 7.20±0.75 | 21.06±1.70 | 56.15±4.57 | 16.58±1.09 |
| OE-27 | 7.21±1.17 | 21.14±1.89 | 56.20±3.71 | 17.59±2.40 |
| OE-30 | 6.82±1.47 | 22.22±2.23 | 52.44±3.76 | 17.01±1.02 |
| OE-S1 | 7.00±0.89 | 22.78±0.68 | 13.51±4.92** | 8.68±1.40** |
| OE-S2 | 7.22±1.33 | 21.68±1.79 | 15.84±2.83** | 9.82±1.42** |
| Ri-2 | 6.61±0.80 | 22.30±1.45 | 10.36±3.32** | 8.02±0.85** |
| Ri-15 | 7.63±1.02 | 20.61±1.96 | 3.62±1.02** | 7.32±0.46** |
| OE-16 | 7.20±1.17 | 21.14±0.89 | 57.60±3.71 | 17.59±2.40 |
| OE-19 | 6.84±1.47 | 22.22±1.23 | 54.44±3.76 | 16.01±1.02 |
| Ri-21 | 7.06+0.89 | 22.78±1.68 | 12.03±4.92** | 8.91±1.40** |
| Ri-23 | 7.21±1.33 | 21.68±0.79 | 9.86±2.83** | 8.33±1.62** |
| OE-1 | 7.62±0.83 | 20.88±1.36 | 68.20±5.41** | 23.35±2.73** |
| OE-2 | 7.01±1.20 | 22.18±0.97 | 76.86±0.86** | 27.06±1.63** |

OE-27 and OE-30 were two independent homozygous from *bZIP73^Jap^* overexpression lines (73^Jap^OE); OE-S1 and OE-S2 were two independent homozygous from *bZIP73^Ind^* overexpression lines (73^Ind^OE); Ri-2 and Ri-15 were two independent homozygous from *bZIP73^Jap^* RNAi lines (73^Jap^Ri); OE-16 and OE-19 were two independent homozygous from *bZIP71* overexpression lines (71OE); Ri-21 and Ri-23 were two independent homozygous *bZIP71* RNAi lines (71Ri). OE-1 and OE-2 were two independent homozygous from co-expression *bZIP71* and *bZIP73^Jap^* lines (71-73^Jap^OE); ZH11: Wild-type Zhonghua11. n=30, **Highly significant difference, P<0.01 level.

# **Table S2** Agronomic traits of *bZIP71* and *bZIP73^Jap^* co-overexpression transgenic lines and wild-type ZH11 grown in the field under natural cold stress conditions for plants heading on September 20, 2012, Beijing.

| Line | Till numbers | Panicle length (cm) | Seed setting rate (%) | Yield per plant (g) |
| --- | --- | --- | --- | --- |
| ZH11 | 6.87±0.54 | 20.43±1.28 | 21.46±3.01 | 12.11±1.73 |
| OE-1 | 6.62±0.78 | 19.89±1.05 | 52.50±4.96** | 16.70±2.03** |
| OE-2 | 7.01±1.32 | 21.07±0.83 | 63.50±3.78** | 18.43±1.74** |

OE-1 and OE-2 were two independent homozygous from co-expression *bZIP71* and *bZIP73^Jap^* lines (71-73^Jap^OE); ZH11: Wild-type Zhonghua 11. n=30, **Highly significant difference, P<0.01 level.

# **Table S3.** Agronomic traits of *bZIP71*, *bZIP73* transgenic lines and wild-type ZH11 grown in the field under normal warm condition for plants heading on August 17, 2012, Beijing.

| Line | Till numbers | Panicle length (cm) | Seed setting rate (%) | Yield per plant (g) |
| --- | --- | --- | --- | --- |
| ZH11 | 23.16±0.71 | 23.16±1.71 | 91.95±2.01 | 32.11±3.93 |
| OE-27 | 23.50±0.42 | 23.50±1.42 | 93.00±3.96 | 33.94±2.06 |
| OE-30 | 23.54±1.07 | 23.54±1.07 | 92.03±2.80 | 33.46±2.77 |
| OE-S1 | 23.64±0.63 | 23.64±1.63 | 89.90±1.79 | 32.16±1.02 |
| OE-S2 | 24.62±0.73 | 24.62±2.73 | 91.31±4.31 | 33.10±3.96 |
| Ri-2 | 23.98±0.84 | 23.98±1.84 | 93.52±2.29 | 31.38±2.29 |
| Ri-15 | 23.84±0.78 | 23.84±0.78 | 89.61±3.08 | 32.37±3.93 |
| OE-16 | 9.60±0.64 | 22.37±1.24 | 92.42±4.96 | 32.44±1.39 |
| OE-19 | 10.40±1.28 | 23.54±1.37 | 93.63±3.80 | 33.46±1.42 |
| Ri-21 | 8.90±1.37 | 23.64±1.63 | 90.30±4.79 | 31.38±2.06 |
| Ri-23 | 9.40±1.86 | 22.07±1.73 | 92.53±3.31 | 32.37±2.53 |
| OE-1 | 8.60±0.49 | 23.50±1.42 | 90.41±2.96 | 31.94±4.34 |
| OE-2 | 10.00±1.26 | 23.54±1.07 | 91.62±4.18 | 32.46±3.44 |

OE-27 and OE-30 were two independent homozygous from *bZIP73^Jap^* overexpression lines (73^Jap^OE); OE-S1 and OE-S2 were two independent homozygous from *bZIP73^Ind^* overexpression lines (73^Ind^OE); Ri-2 and Ri-15 were two independent homozygous from *bZIP73^Jap^* RNAi lines (73^Jap^Ri); OE-16 and OE-19 were two independent homozygous from *bZIP71* overexpression lines (71OE); Ri-21 and Ri-23 were two independent homozygous *bZIP71* RNAi lines (71Ri). OE-1 and OE-2 were two independent homozygous from co-expression *bZIP71* and *bZIP73^Jap^* lines (71-73^Jap^OE); ZH11: Wild-type Zhonghua11. n=30.

# **Table S4.** Agronomic traits of *qLTG3-1^Nip/Ka/Hj^* overexpression transgenic lines and wild-type Kasalath grown in the field under natural cold stress conditions for plants heading on September 3, 2012, Beijing.

| Line | Till numbers | Panicle length (cm) | Seed setting rate (%) | Yield per plant (g) |
| --- | --- | --- | --- | --- |
| Kasalath | 10.10±1.90 | 25.70±0.81 | 42.73±2.81 | 11.88±0.93 |
| N3 | 10.40±1.36 | 25.42±1.54 | 83.69±4.63** | 21.68±2.40** |
| N8 | 9.64±1.85 | 26.5±1.37 | 73.37±3.57** | 18.14±1.02** |
| Ka1 | 9.80±1.90 | 26.02±2.82 | 44.08±3.21 | 12.81±1.24 |
| Ka2 | 10.40±0.83 | 24.5±2.15 | 40.21±3.42 | 12.01±0.74 |
| Hj3 | 10.64±1.85 | 26.16±0.58 | 44.98±3.52 | 11.54±0.64 |
| Hj5 | 9.75±0.95 | 25.34±1.41 | 41.32±2.51 | 12.23±1.32 |

N3 and N8 were two independent homozygous from *qLTG3-1^Nip^* overexpression lines; Ka1 and Ka2 were two independent homozygous from *qLTG3-1^Ka^* overexpression; Hj3 and Hj5 were two independent homozygous from *qLTG3-1^Hj^* overexpression lines. n=30, ** Highly significant difference, P<0.01 level.

# **Table S5.** Agronomic traits of *qLTG3-1^Nip/Ka/Hj^* overexpression transgenic lines and wild-type Kasalath grown in the field under natural cold stress conditions, for plants heading on September 12, 2012, Beijing.

| Line | Till numbers | Panicle length (cm) | Seed setting rate (%) | Yield per plant (g) |
| --- | --- | --- | --- | --- |
| Kasalath | 10.03±1.73 | 25.73±0.73 | 23.98±3.52 | 7.25±0.43 |
| N3 | 10.60±1.84 | 25.68±0.89 | 54.69±3.81** | 14.68±0.32** |
| N8 | 9.87±0.87 | 26.34±1.28 | 43.67±2.87** | 11.24±0.93** |
| Ka1 | 10.04±1.23 | 25.72±2.47 | 24.43±3.52 | 7.41±0.38 |
| Ka2 | 10.21±0.97 | 24.91±2.28 | 21.47±3.81 | 6.81±0.45 |
| Hj3 | 10.56±1.22 | 25.88±1.55 | 23.48±3.12 | 6.93±0.47 |
| Hj5 | 9.84±0.88 | 25.16±1.78 | 21.32±2.96 | 7.26±0.57 |

N3 and N8 were two independent homozygous from *qLTG3-1^Nip^* overexpression lines; Ka1 and Ka2 were two independent homozygous from *qLTG3-1^Ka^* overexpression; Hj3 and Hj5 were two independent homozygous from *qLTG3-1^Hj^* overexpression lines. n=30, ** Highly significant difference, P<0.01 level.

# **Table S6.** Agronomic traits of *qLTG3-1^Nip/Ka/Hj^* overexpression transgenic lines and wild-type Kasalath grown in the field under normal warm conditions for plants heading on August 22, 2012, Beijing.

| Line | Till numbers | Panicle length (cm) | Seed setting rate (%) | Yield per plant (g) |
| --- | --- | --- | --- | --- |
| Kasalath | 10.81±1.72 | 27.50±0.95 | 93.73±2.45 | 24.88±2.77 |
| N3 | 11.10±1.56 | 27.32±1.29 | 94.52±2.52 | 25.65±1.02 |
| N8 | 11.02±1.41 | 26.60±1.42 | 93.91±3.58 | 25.16±2.96 |
| Ka1 | 10.60±1.74 | 26.82±1.01 | 92.89±2.64 | 24.14±1.79 |
| Ka2 | 10.87±1.51 | 27.50±1.24 | 91.37±2.23 | 25.85±1.81 |
| Hj3 | 11.44±1.85 | 27.16±0.58 | 93.98±3.21 | 25.54±2.24 |
| Hj5 | 10.75±1.95 | 26.34±1.41 | 94.12±3.51 | 24.23±1.52 |

N3 and N8 were two independent homozygous from *qLTG3-1^Nip^* overexpression lines; Ka1 and Ka2 were two independent homozygous from *qLTG3-1^Ka^* overexpression; Hj3 and Hj5 were two independent homozygous from *qLTG3-1^Hj^* overexpression lines. n=30, ** Highly significant difference, P<0.01 level.

# **Table S7.** Primers used in real-time PCR analyses and vector constructions.

| **A.** Primers used in real time PCR analysis | | | |
| --- | --- | --- | --- |
| Gene | Forward sequence (5’-3’) | | Reverse sequence (5’-3’) |
| *bZIP73* | TCAGTTCCCACTACCACAGCAACA | | TACCACAGGGAGCAATTCCTGGAT |
| *bZIP71* | TGTGTGCCCTAACTGACATCCTGA | | AAGTCTATGGGTGGCTGGTTCCAT |
| *qLTG3-1* | CCTCCACTTCTTCACCTTCTC | | GCACGTTTATCAGCCCATTC |
| *OsMST7* | GTTCGGGCTCTTCTTCTTCTT | | ATCCTGTCCATCTCCTCGAT |
| *OsMST8* | GATGGCGCTCACAAGAACTA | | GATGTCGTAGCCGAAGATGAG |
| *OsINV4* | GGTCATCAAGAAGGGCAACTAC | | TTGATCGCGAACGCCATATC |
| *Ubiquitin* | GCTCCGTGGCGGTATCAT | | CGGCAGTTGACAGCCCTAG |
| **B.** Primers used in ChIP-qPCR analysis | | | |
| Gene | Forward sequence (5’-3’) | | Reverse sequence (5’-3’) |
| *OsNCED3* | AAGCTGAACAGCAGCCAAG | | CGTGGGCGGTTTGAACG |
| *OsNCED5* | AGTGTCCAAGTCCACTGAG | | AGGAGGAGAGGGAGGAG |
| *qLTG3-1* | CATATTCCCTCCGTCCCAAA | | AAAGACAAGTCGCGCATAAA |
| *OsMST7* | GGTGGATTATGTTATCTCTACCATGT | | CCTGGCTTATTCAAACTAATCTCT |
| *OsMST8* | CTGCAGCAGTAGCACTCTATAA | | GCGTATGTGCCATCTCAA |
| *OsINV4* | CATGGACAAACGGACGGA | | GTCTCGTCTCTCGCCATC |
| *Ubiquitin* | GCTCCGTGGCGGTATCAT | | CGGCAGTTGACAGCCCTAG |
| **C.** **Primers used in EMSA analysis** | | | |
| Gene | | Sequence (5'-3') |  |
| *qLTG3-1^Nip^* | | TTGAAAAAATAATGGGTAGAAAAAACTATCAATTA**TACGTG**ACGAAACATTGAGATATATGTATTAGAGA | |
| *qLTG3-1Nip* (MG) | | TTGAAAAAATAATGGGTAGAAAAAACTATCAATTA**TAAAAG**ACGAAACATTGAGATATATGTATTAGAGA | |
| *OsMST7* | | TTGAAAAAATAATGGGTAGAAAAAACTATCAATTA**TACGTG**ACGAAACATTGAGATATATGTATTAGAGA | |
| *OsMST7*(MG) | | TTGAAAAAATAATGGGTAGAAAAAACTATCAATTA**TAAAAG**ACGAAACATTGAGATATATGTATTAGAGA | |
| *OsMST8* | | TGTACACTGATCTTGTGCTATAGCCTATAG**GACGTG**CCATACAGTACGAAAAATATCATTTTATATTTTC | |
| *OsMST8*(MG) | | TGTACACTGATCTTGTGCTATAGCCTATAG**GAAAAG**CCATACAGTACGAAAAATATCATTTTATATTTTC | |
| *OsINV4* | | GGACGGGCGTCGCTCGCGCGGCGGTT**CACGTG**GCGGCTAGCAGAGCGCCCGCACGCGGGTGGGCGCGAGC | |
| *OsINV4*(MG) | | GGACGGGCGTCGCTCGCGCGGCGGTT**CAAGGC**GCGGCTAGCAGAGCGCCCGCACGCGGGTGGGCGCGAGC | |
| **D.** Primers used in vector construction | | | |
| vectors | Forward sequence (5’-3’) | | Reverse sequence (5’-3’) |
| pTOPO-71 | CACCACAAAATGTCGAGTGGGACCTCGTC | | GAAGCACTGGTACTGGTACAAGTCT |
| pTOPO-73^Jap^ | CACCACAAAATGCTGCACCACCATTACCATGG | | AATATTCT**C**GCATGGCTGTGAGGAG |
| pTOPO-73^Ind^ | CACCACAAAATGCTGCACCACCATTACCATGG | | AATATTCT**T**GCATGGCTGTGAGGAG |
| BD-73^Jap^ | GGAATTCCATATGATGCTGCACCACCATTACCATGG | | CGGAATTCAATATTCTCGCATGGCTGTGAGGAG |
| AD-qLTG3-1^Nip^ | GGAATTCCATATGATGGCGACGAAAGCTGGG | | CGGAATTCGTTGCTGCACTGGAAGCCG |
| AD-qLTG3-1^Nip^N | GGAATTCCATATGATGGCGACGAAAGCTGGG | | CGGAATTCTCTTCCTCCTCCCCCGCC |
| AD-qLTG3-1^Nip/ka^C | GGAATTCCATATGTGCCCGATCGACACGCT | | CGGAATTCGTTGCTGCACTGGAAGCCG |
| AD-qLTG3-1^Ka^ | GGAATTCCATATGATGGCGACGAAAGCTGGG | | CGGAATTCGTTGCTGCACTGGAAGCCG |
| AD-qLTG3-1^Hj^ | GGAATTCCATATGATGGCGACGAAAGCTGGG | | CGGAATTCTTCAAGGTCGGCGAGGCC |
| pJG4-5- *bZIP73^Jap^* | TGCCTCTCCCGAATTCATGCTGCACCACCATTACCATGG | | TCCAAAGCTTCTCGAGAATATTCTCGCATGGCTGTGAGGAG |
| p*qLTG3-1^Nip^*:: LacZ | TATTGGATCGGAATTCAATTGATTTATATTGGGACAGAGACG | | ATGCCTCGAGGTCGACCCCACCCACCGCACTGCA |
| p*OsMST7*:: LacZ | TATTGGATCGGAATTCATCCTATCCAACCCAAGCATCC | | ATGCCTCGAGGTCGACTGCCAGGGAAACGAGAGAAG |
| p*OsMST8*:: LacZ | TATTGGATCGGAATTCTAGTCTGTGTGCCGAGATGAAATA | | ATGCCTCGAGGTCGACGATGGGAATTCCGAAGTGTG |
| p*OsINV4*:: LacZ | TATTGGATCGGAATTCAGGGTTTGCGAGGAGATGG | | ATGCCTCGAGGTCGACTTCCACTAACACACTACTTACCTCGC |
| p1390U- *qLTG3-1^Nip^* | CGGGGTACCATGGCGACGAAAGCTGGG | | CGCGGATCCGTTGCTGCACTGGAAGCCG |
| p1390U- *qLTG3-1^Ka^* | CGGGGTACCATGGCGACGAAAGCTGGG | | CGCGGATCCGTTGCTGCACTGGAAGCCG |
| p1390U- *qLTG3-1^Hj^* | CGGGGTACCATGGCGACGAAAGCTGGG | | CGCGGATCCTTCAAGGTCGGCGAGGCC |
| qLTG3-1^Nip^:: GFP | CGCGGATCCATGGCGACGAAAGCTGGG | | ACGCGTCGACGTTGCTGCACTGGAAGCCG |
| qLTG3-1^Ka^:: GFP | CGCGGATCCATGGCGACGAAAGCTGGG | | ACGCGTCGACGTTGCTGCACTGGAAGCCG |
| p*qLTG3-1^Nip^*:: LUC | CCCCCTCGAGGTCGACAATTGATTTATATTGGGACAGAGACG | | TTGGCGTCTTCCATGGCCCACCCACCGCACTGCA |
| p*OsMST7*::LUC | CCCCCTCGAGGTCGACATCCTATCCAACCCAAGCATCC | | TTGGCGTCTTCCATGGGATGGGAATTCCGAAGTGTG |
| p*OsMST8*::LUC | CCCCCTCGAGGTCGACTAGTCTGTGTGCCGAGATGAAATA | | TTGGCGTCTTCCATGGGATGGGAATTCCGAAGTGTG |
| p*OsINV4*::LUC | CCCCCTCGAGGTCGACAGGGTTTGCGAGGAGATGG | | TTGGCGTCTTCCATGGTTCCACTAACACACTACTTACCTCGC |

BD-73^Jap^: pGBKT7-bZIP73^Jap^; AD-qLTG3-1^Nip^: pGADT7-qLTG3-1^Nip^ (aa:1-184); AD-qLTG3-1^Nip^N: pGADT7-qLTG3-1^Nip^N (aa:1-99); AD-qLTG3-1^Nip/Ka^C: pGADT7 -qLTG3-1^Nip/Ka^C (aa:100-184); AD-qLTG3-1^Ka^: pGADT7-qLTG3-1^Ka^ (aa:1-184); AD-qLTG3-1^Hj^: pGADT7 -qLTG3-1^Hj^(aa:1-116); pTOPO-71: pENTER-TOPO-*bZIP71*; pTOPO-73^Jap^: pENTER-TOPO-*bZIP73^Jap^*; pTOPO-73^Ind^: pENTER-TOPO-*bZIP73^Ind^*; p1390U-qLTG3-1^Nip^: pCambia1390-Ubiquitin-*qLTG3-1^Nip^*; p1390U-qLTG3-1^Ka^: pCambia1390-Ubiquitin-*qLTG3-1^Ka^*; p1390U-qLTG3-1^Hj^: pCambia1390- Ubiquitin-*qLTG3-1^Hj^*; qLTG3-1^Nip^::GFP : pCambia2300-qLTG3-1^Nip^-GFP; qLTG3-1^Ka^::GFP : pCambia2300-qLTG3-1^Ka^-GFP; p*qLTG3-1^Nip^*::LacZ : pLacZi2μ-p*qLTG3-1^Nip^*; p*OsMST7*::LacZ : pLacZi2μ-p*OsMST7*; p*OsMST8*::LacZ : pLacZi2μ-p*OsMST8*; p*OsINV4*::LacZ: pLacZi2μ-p*OsINV4*. p*qLTG3-1^Nip^*::LUC : pGreenII 0800-p*qLTG3-1^Nip^*-LUC; p*OsMST7*::LUC : pGreenII 0800-p*OsMST7*-LUC; p*OsMST8*::LUC : pGreenII 0800-p*OsMST8*-LUC; p*OsINV4*::LUC: pGreenII 0800-p*OsINV4*-LUC.
